# Integrated multi-dimensional analysis highlights DHCR7 mutations involving in cholesterol biosynthesis and contributing therapy of gastric cancer

**DOI:** 10.1186/s13046-023-02611-6

**Published:** 2023-01-30

**Authors:** Yuqi Chen, Wenying Yan, Kexi Yang, Yiting Qian, Yanjun Chen, Ruoqin Wang, Jinghan Zhu, Yuxin He, Hongya Wu, Guangbo Zhang, Tongguo Shi, Weichang Chen

**Affiliations:** 1 Department of Gastroenterology, The First Affiliated Hospital of Soochow University, 188 Shizi Road, Suzhou, 215006 China; 2 Department of Bioinformatics, Center for Systems Biology, School of Biology and Basic Medical Sciences, Medical College of Soochow University, Suzhou, China; 3 Jiangsu Institute of Clinical Immunology, The First Affiliated Hospital of Soochow University, 178 East Ganjiang Road, Suzhou, 215021 China; 4 Jiangsu Key Laboratory of Clinical Immunology, Soochow University, Suzhou, China; 5 Jiangsu Key Laboratory of Gastrointestinal Tumor Immunology, The First Affiliated Hospital of Soochow University, Suzhou, China

**Keywords:** Gastric cancer, GWAS, DHCR7, Mutations, Cholesterol biosynthesis

## Abstract

**Background:**

Genetic background plays an important role in the occurrence and development of gastric cancer (GC). With the application of genome-wide association study (GWAS), an increasing number of tumor susceptibility genes in gastric cancer have been discovered. While little of them can be further applicated in clinical diagnosis and treatment due to the lack of in-depth analysis.

**Methods:**

A GWAS of peripheral blood leukocytes from GC patients was performed to identify and obtain genetic background data. In combination with a clinical investigation, key SNP mutations and mutated genes were screened. Via in vitro and in vivo experiments, the function of the mutated gene was verified in GC. Via a combination of molecular function studies and amino acid network analysis, co-mutations were discovered and further identified as potential therapeutic targets.

**Results:**

At the genetic level, the G allele of rs104886038 in DHCR7 was a protective factor identified by the GWAS. Clinical investigation showed that patients with the rs104886038 A/G genotype, age ≥ 60, smoking ≥ 10 cigarettes/day, heavy drinking and *H. pylori* infection were independent risk factors for GC, with odds ratios of 12.33 (95% CI, 2.10 ~ 72.54), 20.42 (95% CI, 2.46 ~ 169.83), and 11.39 (95% CI, 1.82 ~ 71.21), respectively. Then molecular function studies indicated that DHCR7 regulated cell proliferation, migration, and invasion as well as apoptosis resistance via cellular cholesterol biosynthesis pathway. Further amino acid network analysis based on the predicted structure of DHCR7 and experimental verification indicated that rs104886035 and rs104886038 co-mutation reduced the stability of DHCR7 and induced its degradation. DHCR7 mutation suppressed the malignant behaviour of GC cells and induced apoptosis via inhibition on cell cholesterol biosynthesis.

**Conclusion:**

In this work, we provided a comprehensive multi-dimensional analysis strategy which can be applied to in-depth exploration of GWAS data. DHCR7 and its mutation sites identified by this strategy are potential theratic targets of GC via inhibition of cholesterol biosynthesis.

**Supplementary Information:**

The online version contains supplementary material available at 10.1186/s13046-023-02611-6.

## Background

Gastric cancer (GC) is one of the most lethal and prevalent malignancies worldwide [1]. Notably, its incidence exhibits remarkable geographic heterogeneity, and the highest rates are observed in Asia. Although behavioural risk factors (e.g., smoking [2], alcohol use [3], and diet [4–6]) and infection (e.g., *Helicobacter pylori* (*H. pylori*) infection [7]) are major contributors to GC, genetic susceptibility [8, 9] is still worth noting.

With the application of genome-wide association study (GWAS), an increasing number of tumor susceptibility genes in GC have been discovered. For instance, rs2494938 [10], rs4072037 [11] and rs2294008 [11] (located nearest to LRFN2, MUC1 and PSCA, respectively) are associated with noncardia GC risk in Asia. Hannes Helgason et al. reported loss-of-function variants of ATM (p.Gln852 and p.Ser644) associated with GC risk in a European population and indicated a pathogenic role for the tandem repeat identified in MUC1 [12]. Although a large number of genes and SNPs have been discovered, the application of genetic information screened from GWAS is still limited. Some recent works have made efforts to optimize the application of GWAS in studying the aetiological mechanism of gastric cancer. Feng Bao et al. proposed a new computational framework, termed probabilistic natural mapping (PALM), for performing gene-level association tests [13]. Manon C. W. Slaander et al. conducted a GWAS based on a cohort grouped by anti-*H. pylori* IgG titre to explore functional implications of genetic variations [14]. However, the mechanisms and causal relationships underlying these associations are still poorly understood because the functions of the SNPs have not been thoroughly explored at the gene and protein levels.

**Fig. 1:**
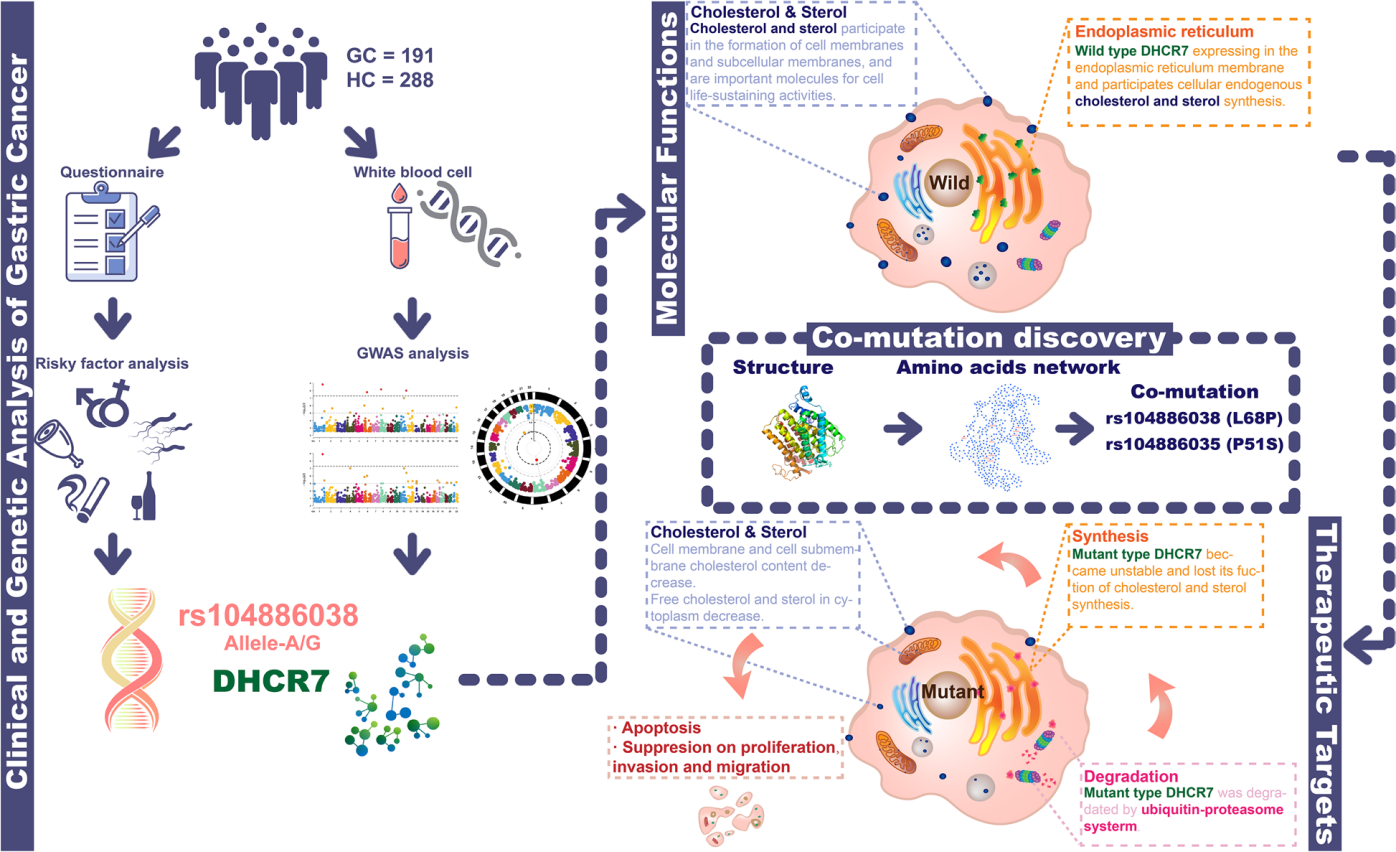
Flowchart of the whole work. In a cohort of 191 GC patients and 288 healthy controls, we conducted a questionnaire survey on lifestyle habits and dietary preferences. To obtain genetic information, peripheral blood leukocytes were collected from patients and controls and used for GWAS. After analysis of genetic and nongenetic risk factors, the DHCR7 gene and its mutation site rs104886038 were identified. Further molecular function verification was carried out in vivo and in vitro experiments. Combined with protein structure analysis, we identified and verified the key co-mutation sites of DHCR7, thereby obtaining potential intervention targets for GC treatment

In this work, we conducted an in-depth study of SNPs in gastric cancer from GWAS data via an integrated multi-dimensional strategy that combines genetic background analysis, clinical investigation, molecular function studies and amino acid network analysis. We identified the G allele of rs104886038, located on DHCR7, as a protective factor against GC by a GWAS. The rs104886038 A/A genotype is an independent risk factor for GC, and the risk of heavy drinking was greatly increased in the group with this genotype. Previous studies have shown that a complex formed by EBP and DHCR7 called cholesterol epoxide hydrolase (CHEH) removes toxic cholesterol epoxides and promotes the growth of tumor cells [15]. Moreover, mutations in DHCR7 are significantly associated with the circulating 25-hydroxyvitamin D concentration and the risk for ovarian cancer [16] and nonmelanoma skin cancer [17]. To evaluate the value of DHCR7 in GC, molecular function studies were performed. DHCR7 knockdown inhibited but DHCR7 overexpression promoted the proliferation, migration and invasion of GC cells in vivo and in vitro. Furthermore, by combined protein structure prediction, amino acid network analysis and functional analysis, we found that DHCR7 mutations impaired the stability of DHCR7 and abolished the promoting effect of DHCR7 on the proliferation, migration and invasion of GC cells. Overall, not only does this knowledge reveal the importance of DHCR7 in gastric cancer, the strategy based on a combination of genetic background analysis, clinical investigation, molecular function studies and amino acid network analysis might also provide new insights for genetic analysis in the post-GWAS era (Fig. 1).

## Methods

### Patients and materials

To perform the GWAS, white blood cell samples from 191 GC patients were obtained as the GC group between April 2018 and May 2019 at the First Affiliated Hospital of Soochow University (Suzhou, China). Samples from 288 volunteers without cancer were collected as the healthy control (HC) group. Table S1 shows the clinicopathological features of the 191 GC patients and baseline information of the healthy controls. The GC patients were staged based on TNM pathological staging criteria (8th edition) established by the American Joint Committee on Cancer (AJCC). We also conducted a questionnaire survey on all the enrolled gastric cancer patients and healthy control volunteers. The contents of the questionnaire including basic information (Gender, Age) and life behaviour (frequency of smoking, frequency of drinking, white meat preference, and pickled food preference). The study protocol was approved by the Institutional Review Board of the First Affiliated Hospital of Soochow University (ethical official number: (2018) No. 016) and was conducted in accordance with the principles of the Declaration of Helsinki. All patients and volunteers were well informed, and written consent was obtained from the study subjects or the legal surrogates of the patients before enrolment.

### DNA extraction and quality control

Ten millilitres of peripheral venous whole blood from all GC and HC participants who had not consumed any food or water was collected into EDTA anticoagulant tubes. After gentle mixing and centrifugation at 4 °C and 1900 × g for 10 min, the upper plasma layer was discarded, and the buffy coat layer was retained as leukocytes. Fifty microlitres of white blood cells was placed into a 1.5 ml centrifuge tube, 500 μl of red blood cell lysate was added, and the tube was vortexed to mix for 10 s and centrifuged at 20,000 × g for 1 min. The waste solution was discarded, and 200 μl of nuclear lysis buffer, 20 μl of proteinase K (20 mg/ml), and 20 μl of SDS (20%) were added and mixed well. After incubation at 60 °C for 20 min, 30 μl of magnetic beads (Beckman XP Beads) was added and mixed by vortexing. Then, 400 μl of absolute ethanol was added and mixed thoroughly. The tube was immediately put on the magnetic frame for 2 min until the liquid was clear, the supernatant was carefully discarded, 1 ml of 80% ethanol was added and mixed for 30 s, the tube was put into the magnetic frame for adsorption for 2 min, and the waste solution was discarded. Then, the tube was dried at room temperature for 5–10 min, and 100 μl of Buffer TE was added for elution, incubated at 56 °C for 5 min, and centrifuged at 20,000 × g for 1 min. The tube was put on a magnetic stand for 2 min, and the supernatant was carefully aspirated. After the gDNA was extracted, quality control was carried out as follows: A Nanodrop spectrophotometer was used to determine the purity of the DNA (OD_260/280_ ratio of 1.8–2.0); agarose gel electrophoresis was performed to analyse the degree of DNA degradation and confirm that there was no obvious degradation, band aggregation without dispersion, or contamination with RNA or proteins; a Qubit fluorometer was then used to accurately quantify the DNA concentration, and DNA samples with an OD value between 1.8 and 2.0 and a total content of more than 100 ng were eligible for use in gene chip detection.

### GWAS genotyping and analysis

GWAS genotyping and analysis were performed by 1Gene, Inc. (Hangzhou, China). First, > 100 ng of leukocyte DNA was quantitatively pipetted into a deep-well plate according to the instructions of the Affymetrix Axiom 2.0 assay. After random amplification, the DNA was fragmented; after thorough mixing, alcohol precipitation was performed; and after drying, the DNA was resuspended in buffer. A small amount was taken for quality control by gel electrophoresis. The fragmented DNA that passed the quality control tests was transferred to a hybridization chip for the high-temperature denaturation and hybridization reactions. After the hybridization reaction was completed, the chip was loaded into an Affymetrix Titan gene chip scanner. The gene chip was customized by Affymetrix. Genotyping data were obtained after staining and signal scanning.

Next, 200 HC and 150 GC samples were randomly selected as the training set, and the remaining 88 HC and 41 GC samples were used as the validation set for the subsequent GWAS. All samples were strictly quality controlled. The detailed quality control and filtration process is shown in Table S2. Briefly, all initial data were filtered according to the following sequence: ① Polymorphic SNP loci; ② Sample data with inconsistent sex information; ③ SNPs on nonautosomal sex chromosomes; ④ SNPs with a genotyping success rate of less than 95%; ⑤ SNPs with a minimum allele frequency of less than 0.05; ⑥ Samples with a genotyping success rate of less than 95% in the samples; ⑦ Samples with a nondesired ancestry; and ⑧ SNPs that were not in Hardy–Weinberg equilibrium. A total of 256,830 SNPs were finally accrued after filtering. Then, the additive model, dominant model and recessive model were performed on the training set. Based on the preliminary analysis results, we finally selected the additive model and applied it to the validation set data to validate the top 5 significant loci in the training set. The SNPs with a *P* value < 0.05 in the validation set were considered significant.

In the training set, we performed all 3 models—the additive model, dominant model and recessive model—to identify the appropriate model. For these 3 models, the numbers of SNPs that we found after data filtering and analysis were 12, 16 and 5, respectively (Table S2). The *P* value threshold was set according to the quantile–quantile plot (Figure S1). Considering the features of GC and the initial results of the GWAS, we finally chose the additive model for further study.

### Differential expression and functional enrichment assay of the TCGA dataset and GEO dataset

RNA-seq data with the corresponding clinical data for 343 GC tissues and 30 adjacent tissues were downloaded from the TCGA database (https://cancergenome.nih.gov/). The DSE66229 dataset was downloaded from the NCBI GEO database (https://www.ncbi.nlm.nih.gov/geo/) and was used for quantification and differential expression analysis of genes with the edgeR package [18] and limma package [19] in R software (4.1.2). Genes with a FC of ≥ 1.5 or ≤ 0.67 and a false discovery rate (FDR) of < 0.05 were considered to be significantly dysregulated between GC tissues and adjacent tissues. The clusterProfiler package [20] in R software was used to perform functional enrichment analysis with the Kyoto Encyclopedia of Genes and Genomes (KEGG) pathway database. All KEGG pathways were filtered with a significance threshold, namely, an adjusted *P* value of < 0.05.

### Protein structure prediction analysis

To study the effects of mutations on protein stability, the structure of DHCR7 was predicted with I-TASSER [21]. The effect of the single mutation L68P (rs104886038) on protein stability was predicted using DynaMut2 [22]. More importantly, we also investigated the effects of the combined L68P mutation with other mutations on protein stability. First, fifty-six mutation sites in DHCR7 were obtained from the UniProt database (https://www.uniprot.org/uniprot/Q9UBM7), and the mutations that were detected in our above SNP microarray were retained for further analysis. Then, the potential co-mutations with L68P were identified as the mutation sites that have a shortest path length smaller than the average shortest path length between L68P (rs104886038) and the remaining residues in the amino acid network. Our previously developed tool ANCA [23] was used to construct the amino acid network of DHCR7 and calculate the shortest path length between residues.

### Cell culture

The cell lines GES-1, AGS, MKN-45, MKN-28 and HGC-27 were obtained from the American Type Culture Collection (ATCC; Manassas, VA, USA). HGC-27 cells were cultured in DMEM (EallBio, Beijing, China, #03.1006C), and the other cells were cultured in RPMI-1640 medium (EallBio, Beijing, China, #03.4007C). The above culture media were supplemented with 10% foetal bovine serum (FBS; EallBio, #03. U16001DC) and 1% penicillin–streptomycin (NCM Biotech, Suzhou, China, #C100C5). All cells were cultured in a humidified incubator with 5% CO_2_ at 37 °C.

### Cell transfection and lentiviral infection

Two commercial nonoverlapping DHCR7 siRNAs (DHCR7-siRNA-1 and DHCR7-siRNA-2) and the control siRNA were purchased from RiboBio (Guangzhou, China). Target sequence of siRNA were provided in Table S3. The expression plasmids carrying wild-type (WT) or mutant (MT) human DHCR7 cDNA and the corresponding control plasmids were purchased from Realgene Biotechnology (Nanjing, China). Cells were transfected with siRNAs or expression plasmids using Lipofectamine 2000 (Invitrogen, Carlsbad, CA, USA) according to the manufacturer’s protocol.

Lentiviruses carrying DHCR7 cDNA or DHCR7 short hairpin RNA (shRNA) containing the sequence of DHCR7-siRNA-1 were purchased from Shanghai GenePharma Co., Ltd. (Shanghai, China). Figure S2A and Figure S2B provided the maps of the shuttle plasmids carrying DHCR7 shRNA or cDNA, respectively. Briefly, 3 packaging plasmids (Figure S2C) and the shuttle plasmid carrying shRNA or cDNA were co-transfected in 293 T cells. After 72 h, the supernatant was collected, centrifuged at 4000 rpm for 4 min at 4 °C. Then, the supernatant was filtered with a 0.45 μm filter, and the filtrate was ultracentrifuged at 4° C, 20,000 rpm for 2 h. The obtained virus concentrate was subjected to titer detection and transfection. An empty backbone vector was used as the control. AGS, MKN-28 and HGC-27 cells in exponential growth phase were grown to 30% confluence and infected with lentiviral particles (MOI: 40). After 72 h, GFP-expressing cells were counted under a fluorescence microscope. Western blotting was used to further confirm the transfection or infection efficiency.

### Western blot analysis

Cells were harvested and lysed in SDS Lysis Buffer (Beyotime, #P0013G) containing protease inhibitors and phosphatase inhibitors (Beyotime, #P1045). Protein concentrations were measured with an Enhanced BCA Protein Assay Kit (Beyotime, #P0010S) according to the manufacturer’s instructions. Total protein (30 μg) was separated by 10% SDS–PAGE (NCM Biotech, Suzhou, China, #P2012) and transferred onto 0.45-µm PVDF membranes (GE Healthcare Life Science, Germany). The membranes were blocked with 5% BSA (Fcmacs, Nanjing, China, #FMS-WB021) for 1.5 h and then incubated with the indicated primary antibodies at 4 °C overnight. The next day, the membranes were incubated with the corresponding HRP-conjugated secondary antibodies for 1 h at room temperature. Finally, the membranes were visualized with ECL reagents (NCM Biotech, Suzhou, China, #10,100) using a ChemiDocTM MP Imaging System (Bio-Rad). All antibodies and their dilution used for western blot analysis were listed in Table S4.

### CCK8 assay

A Cell Counting Kit-8 (NCM Biotech, Suzhou, China, #C6005) was used to evaluate cell proliferation. GC cells from different experimental groups were plated in 96-well plates at a density of 3000 cells per well. Then, 10 μl of CCK8 reagent was added to each well for staining at 37 °C for 2 h. The absorbance at 450 nm was measured.

### EdU incorporation assay

Cells were plated into 48-well plates and cultured overnight. Then, EdU working solution (10 μM, Beyotime, #C0075) was added to the cells. After incubation for 2 h at 37 °C, the cells were fixed with 4% paraformaldehyde and permeabilized with 0.5% Triton X-100 in PBS. Then, the cells were stained with Click Additive Solution according to the manufacturer’s instructions. Nuclei were stained with Hoechst 33,342 (Beyotime, #C1025). Cell counting was subsequently performed under a fluorescence microscope.

### Colony formation assay

GC cells were plated in 12-well plates at a density of 800 cells per well. The cells were cultured at 37 °C for 14 days. After removing the cell culture supernatant, the cells were fixed using 4% paraformaldehyde. Then, the cells were stained with Crystal Violet Staining Solution (Beyotime, #C0121). Images were acquired using an inverted microscope.

### Transwell migration and invasion assays

For the migration assay, 4 × 10^4^ GC cells in 300 μl of serum-free medium were plated in the upper compartments of a 24-well plate containing an 8-μm pore size membrane (Corning, #353097), and 500 μl of medium containing 20% serum was added to the bottom compartments. For the invasion assay, Matrigel (diluted 1:10; Corning, #356234) was used to coat the membrane in the upper compartment. After culture at 37 °C for 48 h, the cells attached to the lower surface of the membrane in the upper compartment were fixed with 4% paraformaldehyde and stained with Crystal Violet Staining Solution. Images were acquired using an inverted microscope.

### Cholesterol measurement

The cholesterol levels in cells or tissues were measured using a total cholesterol assay kit (Jiancheng, Nanjing, China, #A111-1–1) following the manufacturer’s instructions. Briefly, 1 × 10^7^ cells were digested and resuspended in 100 μl of PBS containing 1% Triton X-100. After lysis on ice for 45 min, the cells were centrifuged at 4 °C and 4000 rpm for 10 min. The supernatant was collected for cholesterol measurement. To measure the tissue cholesterol, the tumor tissue was mixed 1:9 by weight and volume with PBS solution containing 1% Triton X-100 and was homogenized. After centrifuging at 4000 rpm, 4 °C for 10 min, the supernatant to taken for the determination of cholesterol. To measure the cholesterol level, 2.5 μl of cell supernatant was added to 250 μl of working solution per well in a 96-well plate. In addition, ddH_2_O and the calibrator were added to the blank and calibrator wells, respectively. After incubation at 37 °C for 10 min, the absorbance at 510 nm was measured. Protein concentrations were calculated using the following equation: $Cholesterol\;concentration=\left(\left({OD}_{sample}-{OD}_{blank}\right)\right)/\left(\left({OD}_{calibrator}-{OD}_{blank}\right)\right)\times calibrator\;concentration\;\div\;protein\;concentration\;in\;sample$. 

### Filipin III staining

GC cells were plated in 48-well plates at a density of 2 × 10^4^ cells per well and cultured at 37 °C overnight. After fixation using 4% paraformaldehyde, the cells were stained with Filipin III (Sigma, St. Louis, USA, #SAE0088) at room temperature (RT) for 2 h. The stained cells were imaged using an inverted microscope. All images were processed using ImageJ software [24]. Briefly, individual cells were segmented on the images according to the GFP signal. First, an intensity threshold was applied to determine the cell contours. A watershed algorithm (find maxima) was used to divide the image into discrete areas. Then, the thresholded and segmented images were merged to generate a mask showing the contours of individual cells. A threshold was applied to the filipin staining image to decrease the background signal and to focus the analysis on bright filipin puncta corresponding to cholesterol accumulation. The segmentation mask was applied to this thresholded image, and the filipin signal intensity was measured in each cell.

### Apoptosis assay

The apoptosis rate was determined by a PE Annexin V Apoptosis Detection Kit I (BD Biosciences, #559763). Cells were collected and washed with cold PBS. Then, the cells were resuspended in 1 × Binding Buffer at a concentration of 1 × 10^6^ cells/ml. Then, 5 µl of Annexin V-PE and 5 µl of 7-AAD were added to the cell suspension. After incubation for 15 min at RT in the dark, apoptosis was analysed by flow cytometry. Annexin-V + /7-AAD- cells and Annexin-V + /7-AAD + cells were considered apoptotic cells.

### Xenograft tumor model

The animal experiments were approved by the Institutional Animal Care and Use Committee of Soochow University (Suzhou, China). Five-week-old female BALB/c nude mice and NSG mice were purchased from the Shanghai Laboratory Animal Center (Shanghai, China). BALB/c nude mice were randomly assigned to the OE-DHCR7 group or the OE-NC group (*n* = 5 per group). To establish the xenograft tumor model, 1 × 10^7^ DHCR7-overexpressing MKN-28 cells or empty vector MKN-28 cells were injected subcutaneously into the right flanks of the mice in the OE-DHCR7 group or the OE-NC group, respectively. The xenograft tumors were measured every 3 days by using digital callipers, and tumor volumes were calculated. After 2 weeks, all mice were sacrificed, and tumor masses were analysed. In addition, the xenograft tumor tissues were used for cholesterol detection and immunohistochemistry (IHC).

In rescue assays with inhibitors targeting cholesterol biosynthesis pathway and DHCR7, NSG mice were randomly assigned to the OE-DHCR7 group and tamoxifen (TAM) treatment (OE-DHCR7-TAM) group (*n* = 5 per group). 1 × 10^7^ DHCR7-overexpressing MKN-28 cells were injected subcutaneously into the right flanks of the mice. The xenograft tumors were measured every 3 days by using digital callipers, and tumor volumes were calculated. After 2 weeks, mice in OE-DHCR7-TAM were administrated TAM (10 mg/kg, i.g.) once a day, for 7 consecutive days. At the same time, mice in OE-DHCR7 were were administrated an equal dose of solvent (corn oil). All mice were sacrificed on the day 28 after cell injection, and tumor masses were analyzed as above.

### Murine lung metastasis model

Five-week-old female NOD/SCID mice and NSG mice were purchased from the Shanghai Laboratory Animal Center (Shanghai, China). NOD/SCID mice were randomly assigned to the OE-DHCR7 group or the OE-NC group (*n* = 4 per group). Mice were injected with DHCR7-overexpressing MKN-28 cells or empty vector MKN-28 cells (3 × 10^6^ cells/200 μl PBS per mouse) via the tail vein. 6 weeks after injection, mice were sacrificed and the lungs were excised and fixed with 4% paraformaldehyde, and stained with hematoxylin and eosin (HE). Lung metastatic nodules were counted in a blinded manner by two experienced pathologists. In rescue assays with inhibitors targeting cholesterol biosynthesis pathway and DHCR7, NSG mice were randomly assigned to the OE-DHCR7 group and the OE-DHCR7-TAM group (*n* = 5 per group). All mice were injected with DHCR7-overexpressing MKN-28 cells (3 × 10^6^ cells/200 μl PBS per mouse) via the tail vein. After 2 weeks, mice in OE-DHCR7-TAM were administrated TAM (10 mg/kg, i.g.), once a day, for 7 consecutive days. At the same time, mice in OE-DHCR7 were administrated an equal dose of solvent (corn oil). 6 weeks after injection, all mice were sacrificed and the lung tissues were obtained for metastatic nodules caculation and HE staining.

### Statistics

All statistical analyses and graphing in this paper were performed with GraphPad Prism (9.3.0) and R software (4.1.2). For normally distributed data, Student’s t test was used, while for nonnormally distributed data, the Wilcoxon rank-sum test was used. Regression analysis was based on a logistic regression model. A *P* value < 0.05 was considered statistically significant.

## Results

### rs104886038 and DHCR7 play an important role in GC

**Fig. 2:**
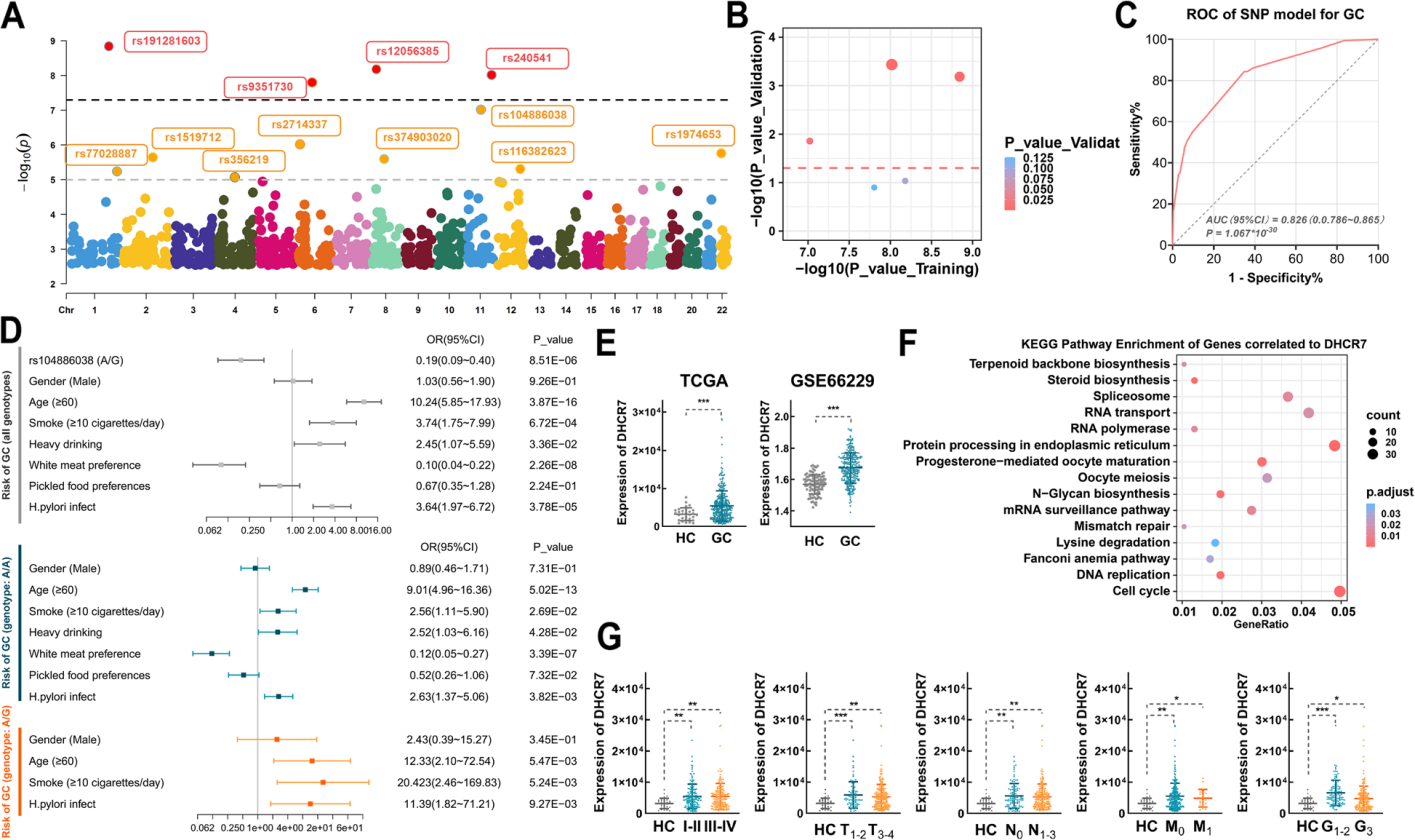
rs104886038 and DHCR7 play important roles in GC. **A** Manhattan plot for the GWAS based on the analysis of the training set with the additive model. **B** Significant SNPs confirmed in the validation set. **C** ROC of the SNP model for GC diagnosis. **D** Forest plots for risk factors for GC. OR(95%CI) and *P* value of OR were calculated based on the logistic regression model. **E** In the TCGA and GSE66229 datasets, DHCR7 was upregulated in GC tissues compared with normal tissues (***FDR < 0.001). **F** The results of functional enrichment analysis of genes coexpressed with DHCR7. **G** The expression of DHCR7 in GC tissues with different pathological TNM stages, primary tumor invasion depths, lymph node metastasis statuses, distant metastasis statuses and differentiation grades (calculated by Student’s t test, *P < 0.05; **P < 0.01; ***P < 0.001)

Consistent with the conventional GWAS analysis approach, we combined the training and validation sets for analysis to identify gene-level changes associated with GC. In the training set analysed with the additive model, there were 12 SNPs that were significantly different between the GC and HC groups when the *P* value threshold was set at 1 × 10^–5^, of which 5 had a *P* value of less than 1 × 10^–7^ (Fig. 2A). The top 5 significant SNPs in the training set were calculated in the validation set. Finally, three of these 5 SNPS – rs191281603, rs240541 and rs104886038 – were selected at *P* < 0.05 level (Fig. 2B). Further annotation was conducted to explore these key SNPs (Table 1). The results showed that the minor allele of rs191281603 (G) was the GC risk genotype, with OR (95% CI) = 7.501 (4.645 ~ 12.113). The minor allele of rs104886038 (G) seemed to be protective, with OR (95% CI) = 0.243 (0.147 ~ 0.404). rs104886038 is located in the coding sequence of DHCR7. Thus, we suspected that DHCR7 is an important gene in GC, and its mutation may be associated with susceptibility. Then, we incorporated all these SNPs into a logistic regression model and calculated the diagnostic efficiency of the model (Table S5, Fig. 2C). Comprehensive evaluation of these SNP genotype characteristics can effectively diagnose GC (AUC = 0.826, *P* = 1.067 × 10^–30^). Then, we evaluated the risk of the rs104886038 genotype together with other common predisposing factors in the logistic regression model. The results showed that the G allele of rs104886038 was a protective factor (OR (95% CI = 0.194 (0.094 ~ 0.400)), *P* = 8.51 × 10^–6^), while other factors, i.e., age ≥ 60, smoking ≥ 10 cigarettes/day, heavy drinking and *H. pylori* infection, were risk factors (Fig. 2D). In patients with the rs104886038 A/G genotype, age ≥ 60, smoking ≥ 10 cigarettes/day, heavy drinking and *H. pylori* infection were independent risk factors for GC, with odds ratios of 12.33 (95% CI, 2.10 ~ 72.54), 20.42 (95% CI, 2.46 ~ 169.83), and 11.39 (95% CI, 1.82 ~ 71.21), respectively. For patients with the A/A genotype, in addition to the risk factors above, the risk of heavy drinking, with an odds ratio of 2.52 (95% CI, 1.03 ~ 6.16), deserves attention (Fig. 2D).

**Table 1:** Germline mutations identified by GWAS analysis

**Chr.**^**a**^	**Variant**	**Major/Minor Allele**	**Associated Gene**	**Case**^**b**^	**Control**^**b**^	**MAF**^**c**^		***P*_value**^**d**^	**OR (95% CI)**	***P*_value**
						**Case**	**Control**			
1:196989521	rs191281603	C/G	CFHR5	102/83/3	258/29/0	0.237	0.051	2.49E-12	7.501 (4.645 ~ 12.113)	7.47E-19
11:126339488	rs240541	A/G	DCPS	46/94/29	13/117/157	0.450	0.751	3.00E-11	0.546 (0.228 ~ 1.312)	1.74E-01
11:71444111	rs104886038	A/G	DHCR7	169/22/0	185/99/0	0.058	0.174	5.74E-09	0.243 (0.147 ~ 0.404)	1.04E-08

^a^Based on GRCh38

^b^Major homozygote/heterozygote/minor homozygote

^c^MAF, minor allele frequency

^d^*P*_value of GWAS based on the combination of the training set and validation set

Next, we compared the expression of DHCR7 between GC tumor tissue and adjacent tissue in a public dataset. In both the TCGA (343 GC vs. 30 HC samples) and GSE66229 (300 GC vs. 100 HC samples) datasets, DHCR7 was significantly upregulated in tumor tissues (Fig. 2E). Functional enrichment analysis showed that genes correlated with DHCR7 (|r |≥ 0.3, *P* < 0.05) were highly enriched in terms such as cell cycle, processing in endoplasmic reticulum, steroid biosynthesis, and DNA replication (Fig. 2F). DHCR7 was consistently upregulated independent of stage, invasion depth, metastasis status, and differentiation grade (Fig. 2G), indicating that it might play a consistently important role in the occurrence and progression of GC.

### DHCR7 knockdown promoted proliferation, migration and invasion in GC cell lines

**Fig. 3:**
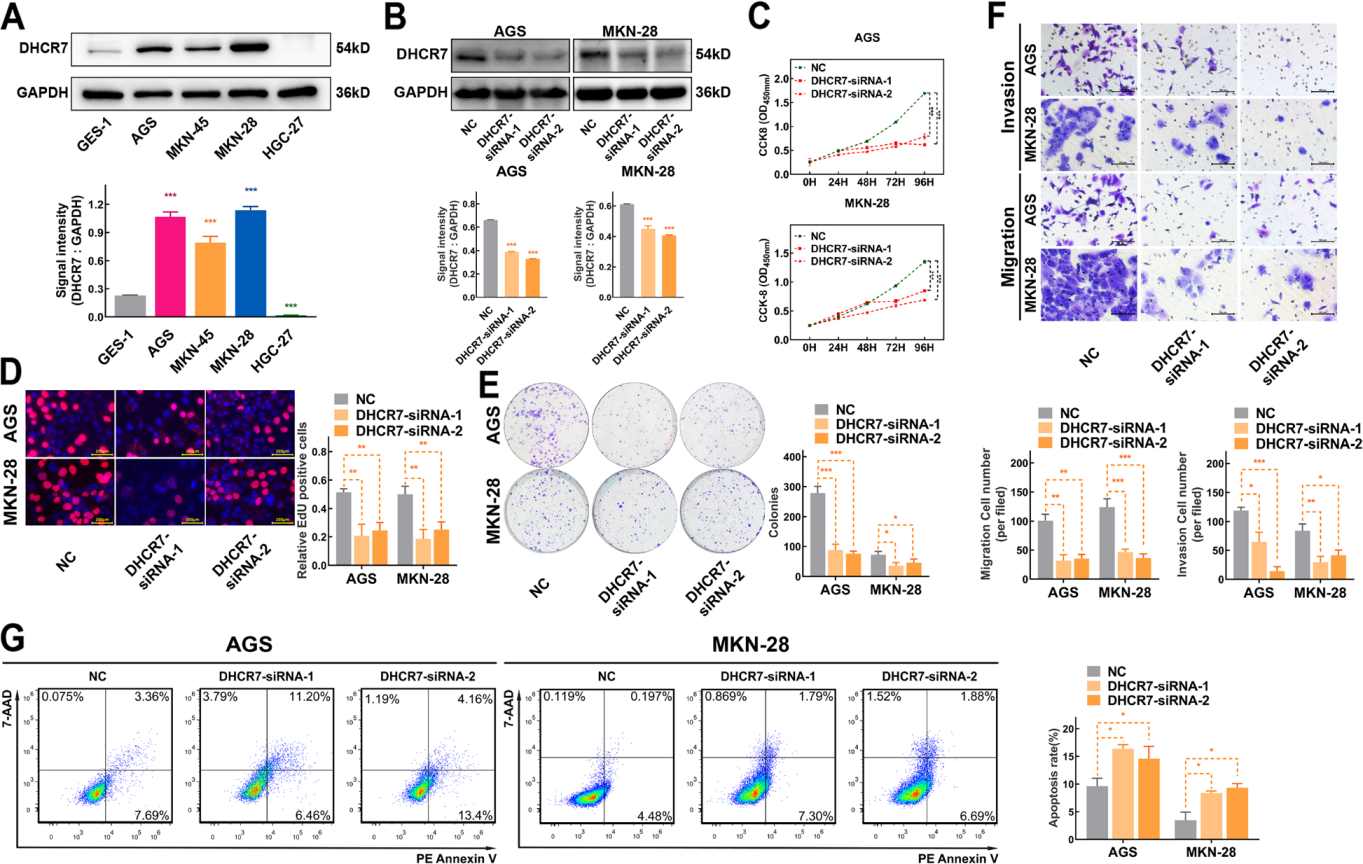
Knockdown of DHCR7 inhibited the proliferation, invasion and migration of GC cells and promoted apoptosis. **A** Protein expression of DHCR7 was signicicantly upregulated in AGS, MKN-45, MKN-28 and downregulated in HGC-27 cells. **B** Western blot analysis confirmed that DHCR7 expression was knocked down by siRNAs in GC cell lines. **C** Proliferation of AGS and MKN-28 cells with different DHCR7 expression levels, as determined by a CCK-8 assay at different time points over a 96 h period. **D** Results of EdU incorporation assays in AGS and MKN-28 cells transfection with DHCR7 siRNAs. **E** Results of colony formation assays in AGS and MKN-28 cells transfection with DHCR7 siRNAs. **F** Results of Transwell migration and invasion assays in AGS and MKN-28 cells transfection with DHCR7 siRNAs. **G** Results of the apoptosis assay in AGS and MKN-28 cells transfection with DHCR7 siRNAs. The data are presented as the means ± SDs and calculated by Student’s t test. *P < 0.05; **P < 0.01; ***P < 0.001

To evaluate and utilize the SNP found in the GWAS, we first conducted a preliminary study on the role of DHCR7, encoded by the gene in which rs104886038 is located. To further investigate the biological function of DHCR7, we analysed the expression of DHCR7 in 4 GC cell lines. As shown in Fig. 3A, the protein level of DHCR7 was significantly increased in AGS, MKN-45 and MKN-28 cells but decreased in HGC-27 cells compared to GES-1 cells. Then, we silenced DHCR7 expression in AGS and MKN-28 cells, which expressed relatively high levels of DHCR7, and the protein level of DHCR7 was obviously decreased after knockdown (Fig. 3B). The CCK8 and EdU incorporation assays showed that DHCR7 knockdown markedly decreased the proliferation rate of AGS and MKN-28 cells (Fig. 3C, D). These results were further supported by a colony formation assay (Fig. 3E). To test the effects of DHCR7 expression on migration and invasion, Transwell assays were performed, and the results confirmed that DHCR7 knockdown suppressed the migration and invasion of AGS and MKN-28 cells (Fig. 3F). Moreover, silencing DHCR7 led to a significant increase in the apoptosis rate of AGS and MKN-28 cells (Fig. 3G).

### DHCR7 overexpression promoted the malignant phenotype of GC by increasing the cholesterol level in GC cells

**Fig. 4:**
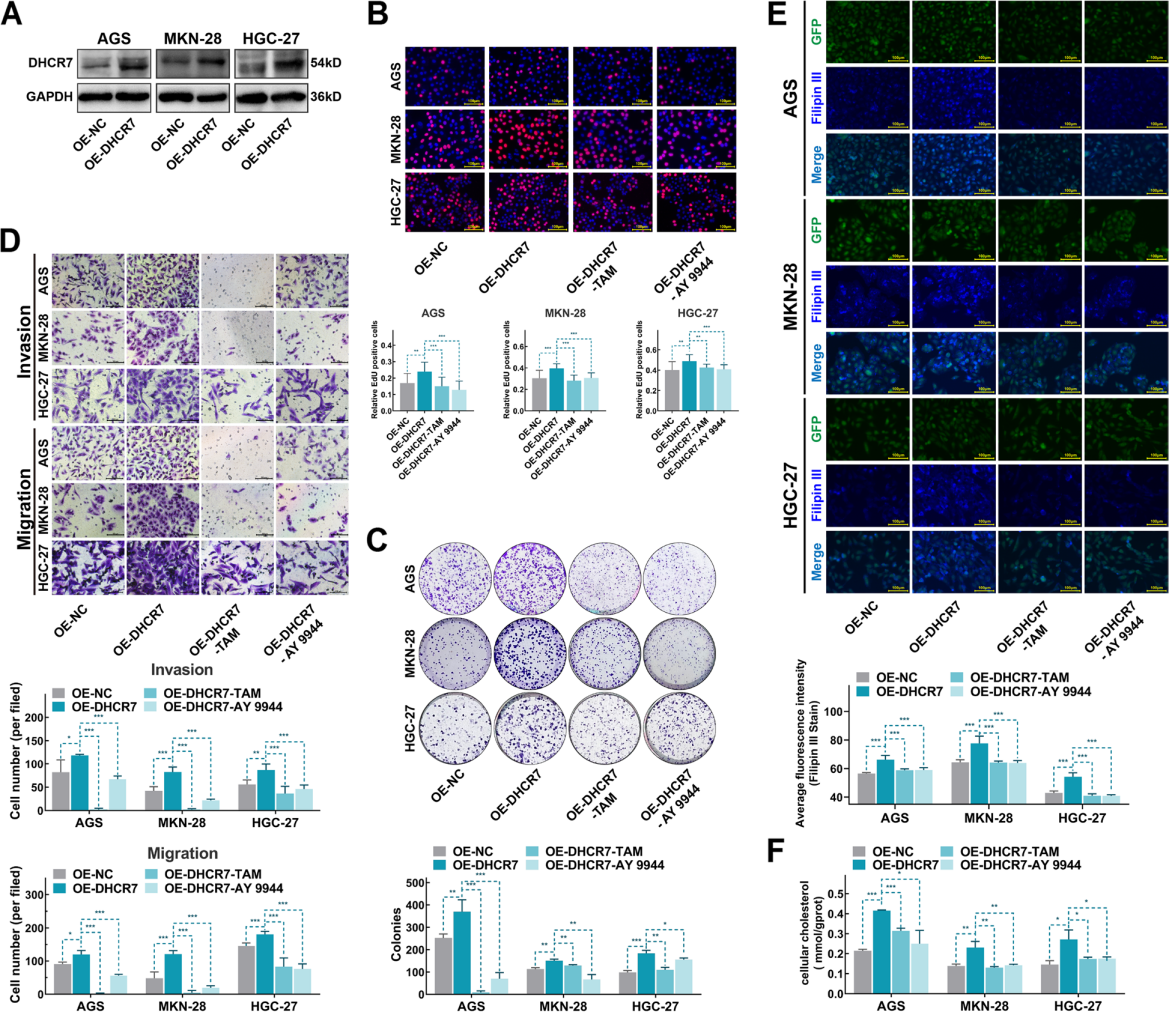
Overexpression of DHCR7 promotes the proliferation, invasion and migration of GC cells via cellular cholesterol biosynthesis. **A** Western blot analysis verified that DHCR7 was stably overexpressed in GC cell lines infected with DHCR7 overexpression lentivirus. **B** Results of EdU incorporation assays in control GC cells, DHCR7-overexpressing GC cells and DHCR7-overexpressing GC cells treated with TAM or AY 9944 for 24 h. **C** Results of colony formation assays in control GC cells, DHCR7-overexpressing GC cells and DHCR7-overexpressing GC cells treated with TAM or AY 9944 for 14 days. **D** Results of Transwell migration and invasion assays in control GC cells, DHCR7-overexpressing GC cells and DHCR7-overexpressing GC cells treated with TAM or AY 9944 for 24 h. **E** Cellular cholesterol level in control GC cells, DHCR7-overexpressing GC cells and DHCR7-overexpressing GC cells treated with TAM or AY 9944 for 24 h, as measured by Filipin III staining. **F** Cholesterol level in control GC cells, DHCR7-overexpressing GC cells and DHCR7-overexpressing GC cells treated with TAM or AY 9944 for 24 h. AGS were treated with TAM (3 μM) and AY 9944 (5 μM). MKN-28 were treated with TAM (5 μM) and AY 9944 (10 μM). HGC-27 were treated with TAM (5 μM) and AY 9944 (5 μM). The data are presented as the means ± SDs and calculated by Student’s t test. *P < 0.05; **P < 0.01; ***P < 0.001

We further examined the biological significance of DHCR7 in GC cells by stably overexpressing cells. AGS, MKN28, and HGC27 cell lines with stable DHCR7 overexpression were established via lentiviral transduction (Fig. 4A). Compared to the corresponding control cells, DHCR7-overexpressing AGS, MKN28, and HGC27 cells exhibited significant increases in the proliferation, clonogenic, migration and invasion abilities (Fig. 4B-D). Moreover, with inhibition by TAM and AY 9944, which were reported to target on cholesterol biosynthesis pathway and DHCR7 [25], the promotion of DHCR7 on malignant phenotype failed to act(Fig. 4B-D, Figure S3). These findings suggested that DHCR7 plays a crucial role in mediating the proliferation, migration and invasion of GC cells. Considering that DHCR7 is an important enzyme involved in cholesterol biosynthesis [26], we examined the cholesterol level in GC cells. As shown in Fig. 4E-F, cellular cholesterol level was increased in DHCR7-overexpressing AGS, MKN28, and HGC27 cells, while both TAM and AY 9944 could significantly decrease the cellular cholesterol level in DHCR7-overexpressing GC cells. These findings indicate that DHCR7 paticipates cell cholesterol biosynthesis pathway to regulate malignant phenotype of GC.

**Fig. 5:**
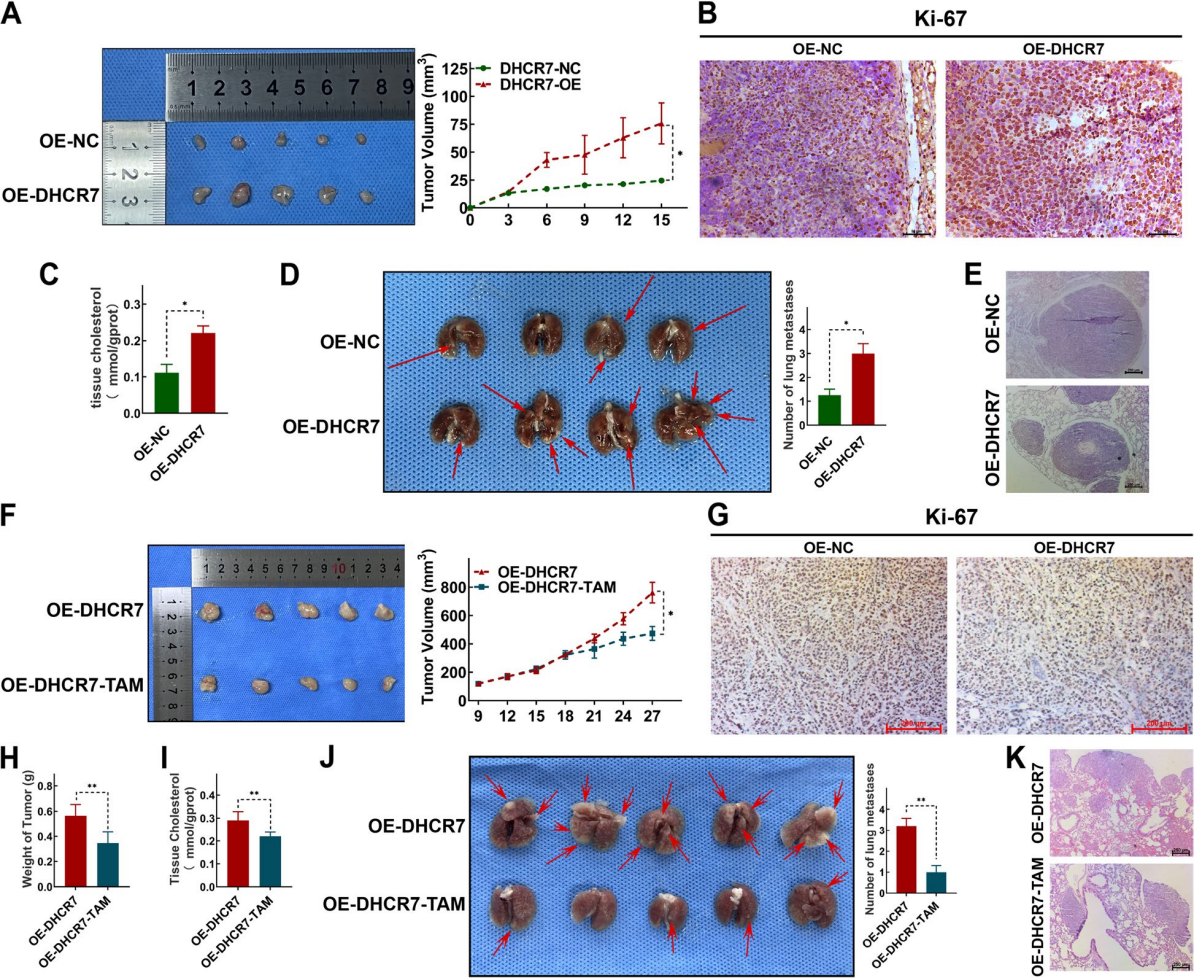
Overexpressing DHCR7 increased the cholesterol level and further promoted the malignant phenotype of GC in vivo. **A** Results of xenograft tumor model generated with DHCR7-overexpressing GC cell and control GC cell (*n* = 5 per group). **B** Representative images of IHC for Ki-67 in tumor tissues from OE-DHCR7 and OE-NC groups. **C** Results of tissue cholesterol detection in tumors from OE-DHCR7 and OE-NC groups. **D** Results of murine lung metastasis model generated with DHCR7-overexpressing GC cell and control GC cell (*n* = 4 per group). **E** Representative images of HE stains in lung metastasis from OE-DHCR7 and OE-NC groups. **F** Results of TAM treatment on DHCR7-overexpressing GC cell in xenograft tumor model (*n* = 5 per group). **G** Representative images of IHC for Ki-67 in tumor tissues from OE-DHCR7 and OE-DHCR7-TAM groups. **H** Weight of tumors from OE-DHCR7 and OE-DHCR7-TAM groups. **I** Tissue cholesterol level of tumors from OE-DHCR7 and OE-DHCR7-TAM groups. **J** Results of TAM treatment on DHCR7-overexpressing GC cell in murine lung metastasis model (*n* = 5 per group). **K** Representative images of HE stains in lung metastasis from OE-DHCR7 and OE-DHCR7-TAM groups. The data represent the means ± SEMs and calculated by Student’s t test. *P < 0.05; **P < 0.01; ***P < 0.001

To assess the effect of DHCR7 on GC tumor growth in vivo, a xenograft mouse model was established using DHCR7-overexpressing MKN28 cells. The tumor images and tumor volumes showed that DHCR7 overexpression significantly promoted tumor growth in vivo (Fig. 5A). Moreover, the expression of Ki67, a proliferation marker, was obviously up-regulated in xenograft tumor tissues from DHCR7 overexpressed group compared with the control group (Fig. 5B). Additionally, the tissue cholesterol levels in DHCR7 overexpressed group were higher than that in the control group (Fig. 5C). To determine whether DHCR7 contributes to the metastasis of GC in vivo, we further established a mouse model of GC lung metastasis using DHCR7-overexpressing cells. As shown in Fig. 5D and E, DHCR7 overexpression markedly enhanced the ability of these cells to form lung metastases in mice. The number of metastatic nodules was significantly increased in the OE-DHCR7 group. To verify whether DHCR7 regulated tumor growth and metastasis of GC in vivo via cholesterol biosynthesis pathway, TAM was used.

Compared with the OE-DHCR7 group, the growth of tumors was markedly suppressed in the OE-DHCR7-TAM group (Fig. 5F-H). The tissue cholesterol levels in the OE-DHCR7-TAM group were significantly lower than that in the OE-DHCR7 group (F ig. 5I). Moreover, the number of lung metastatic nodules was significantly reduced in the the OE-DHCR7-TAM group compared to the OE-DHCR7 group (Fig. 5J-K). Collectively, these results indicated that overexpressing DHCR7 increased the cholesterol level and further promoted the malignant phenotype of GC in vivo.

### Amino acid network-based analysis predicted key mutation sites affecting the stability of DHCR7

**Fig. 6:**
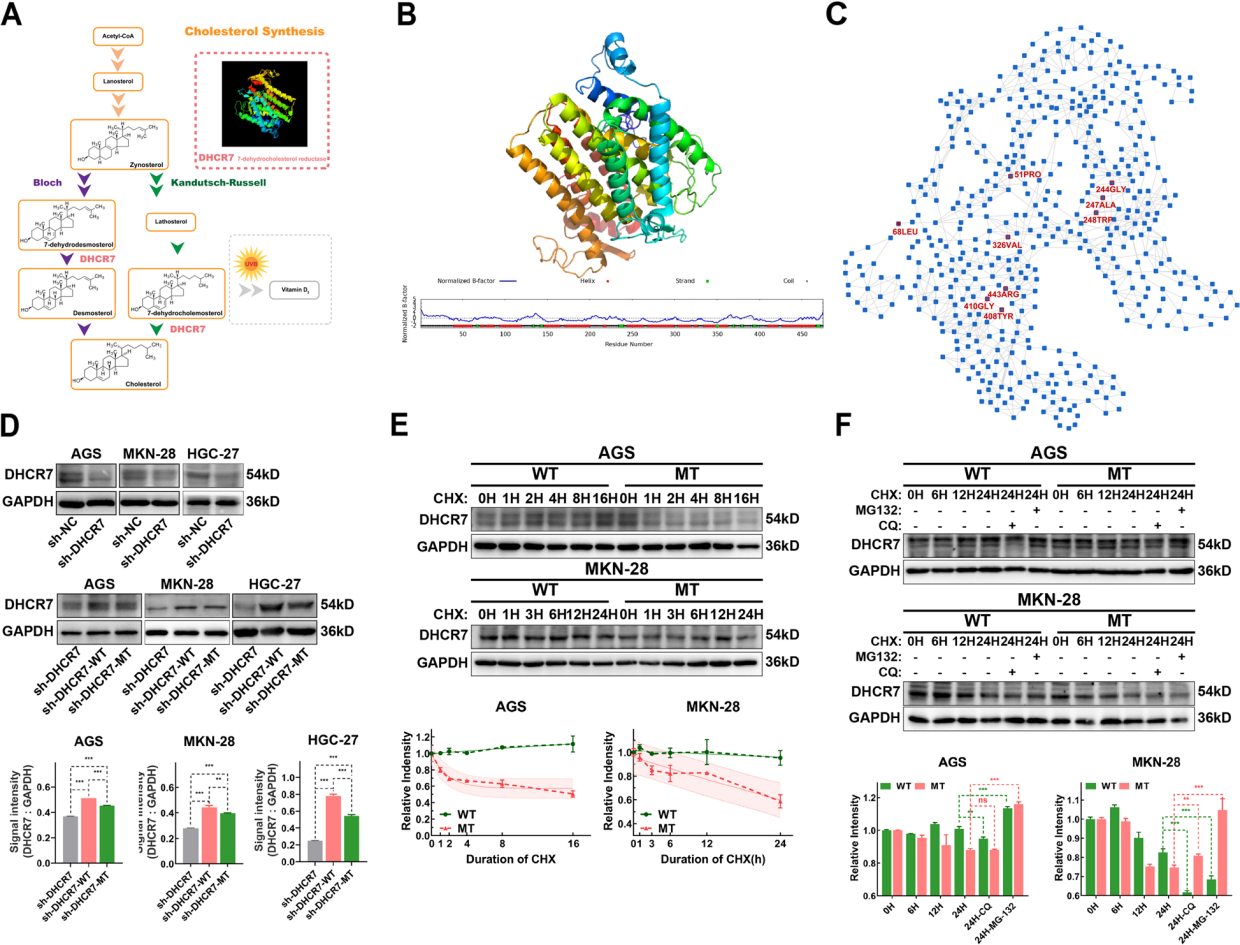
Co-mutation of rs104886038 and rs104886035 in DHCR7 reduces its stability and induces its degradation through the ubiquitin–proteasome system. **A** DHCR7 catalyses the synthesis of cholesterol via both the Bloch and Kandutsch-Russell pathways. **B** Prediction of the structure of DHCR7. **C** Amino acid network of DHCR7. **D** Western blot analysis verified that both wild-type and mutant DHCR7 were overexpressed in sh-DHCR7 GC cells, while the protein expression of mutant DHCR7 was significantly lower than the wild-type. Both wild-type and mutant DHCR7 plasmid were transfected to sh-DHCR7 GC cells. Western blot analysis were performed at 72 h after transfection. **E** CHX (30 μM) continued to exert effects for 16–24 h, and the level of DHCR7 in the different groups was verified by western blot analysis. **F** After CHX treatment, WT/MT GC cell lines were treated with CQ (20 μM) and MG132 (10 μM) to verify the degradation pathway of DHCR7. The data are presented as the means ± SDs and calculated by Student’s t test. *P < 0.05; **P < 0.01; ***P < 0.001

Although previous studies have reported the role of DHCR7 in cholesterol biosynthesis [26], its protein structure still remained unclarified (Fig. 6A). This also limited further exploration of the mechanism of action of DHCR7 and its SNP mutations. After clarifying the importance of DHCR7 in regulating cholesterol biosynthesis in GC cells, we further explored the effects of mutations on DHCR7 protein structure and function to identify potential applications in GC. First, the structure of DHCR7 was predicted, as shown in Fig. 6B. Then, the effects of the L68P (rs104886038) mutation on protein stability were predicted, and the results showed that L68P reduced DHCR7 protein stability (ΔΔG = -0.36 kcal/mol). Since the combined effects of mutations may play a more important role than those of single mutations in cancer, we further investigated the combined effects of L68P and other mutations on protein stability. Based on the amino acid network model and network theory (Fig. 6C), we selected the mutation sites that had a shortest path length to L68 smaller than the average shortest path length between L68 and the remaining residues in the amino acid network as the potential co-mutations with L68P. Eight mutations were selected as the potential co-mutations with L68P, and the effects of the co-mutation pairs on protein stability were predicted. As shown in Table 2, DHCR7 with L68P and P51S co-mutation had the smallest ΔΔG value (-2.31 kcal/mol), and this co-mutation had a larger impact on the reduction in protein stability than did the single L68P mutation.

**Table 2:** The effects of the single L68P mutation and co-mutations with L68P on DHCR7 protein stability

**Co-mutation pair**	**Multiple Mutation ΔΔG**	**Single Mutation ΔΔG** **(L68P: -0.36 kcal/mol)**
L68P + P51S	-2.9	-2.31
L68P + G244R	-0.59	-0.86
L68P + A247V	0.37	0.87
L68P + W248C	-1.91	-2.2
L68P + V326L	-1.16	-0.37
L68P + Y408H	-0.72	-1.57
L68P + G410R	-1.59	-0.77
L68P + R443C	-1.66	-1.24

To verify our predictions, we constructed wild-type and mutant DHCR7 plasmids and transfected them into GC cells with stable DHCR7 knockdown (Fig. 6D). The protein expression level of DHCR7 in DHCR7 mutant (MT) GC cells was lower than that in DHCR7 wild-type (WT) cells (Fig. 6D). In the presence of the protein synthesis inhibitor cycloheximide (CHX), DHCR7 with rs104886038 and rs104886035 co-mutation exhibited a faster turnover rate than WT DHCR7 in AGS and MKN-28 cells (Fig. 6E). To characterize the degradation pathways of DHCR7, the autophagy–lysosome system inhibitor chloroquine (CQ) and the ubiquitin–proteasome system inhibitor MG-132 were used in combination with CHX. As shown in Fig. 6F, the degradation of both WT and mutant DHCR7 proteins was inhibited by MG-132 treatment but was not affected by CQ treatment. These results suggested that DHCR7 with rs104886038 and rs104886035 co-mutation are less stable and presumably more susceptible to degradation through the ubiquitin–proteasome system.

### Mutant DHCR7 suppressed proliferation, invasion, and migration and induced apoptosis in GC cell lines

**Fig. 7:**
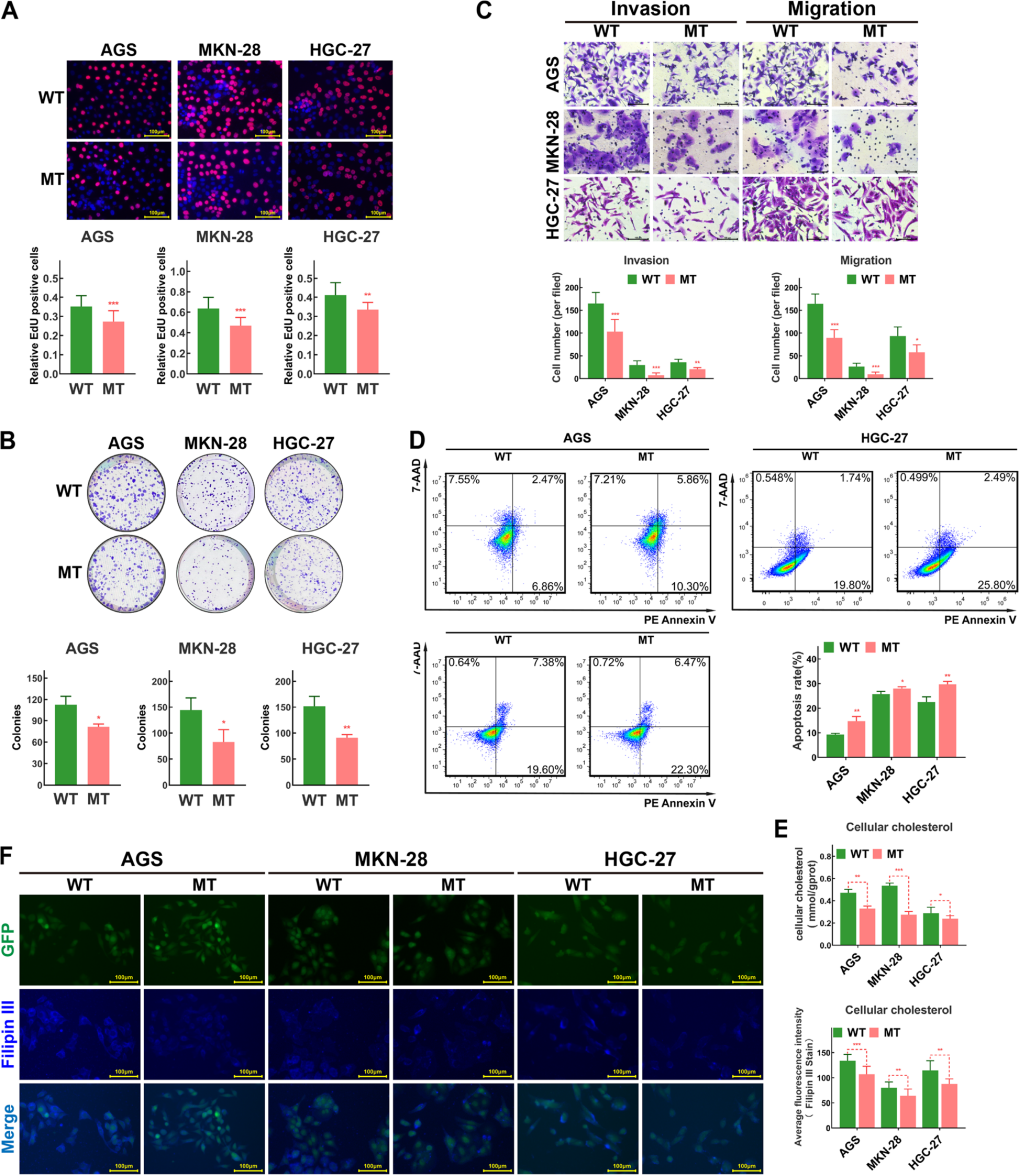
Mutant DHCR7 inhibited the proliferation, invasion and migration of GC cells and promoted apoptosis. **A** The proliferation of GC cells with WT/MT DHCR7 was detected by an EdU incorporation assay. **B** Results of colony formation assays in GC cells with WT/MT DHCR7. **C** Results of Transwell migration and invasion assays in GC cells with WT/MT DHCR7. **D** Results of the apoptosis assay in GC cells with WT/MT DHCR7. **E** Cholesterol level in GC cells with WT/MT DHCR7. **F** Cellular cholesterol level, as measured by Filipin III staining. The data are presented as the means ± SDs and calculated by Student’s t test. *P < 0.05; **P < 0.01; ***P < 0.001

We next investigated the effect of rs104886038 and rs104886035 co-mutation on the biological significance of DHCR7 in GC cells. The results of the EdU incorporation and colony formation assays showed that compared with that in AGS, MKN28, and HGC-27 cells transfected with the WT DHCR7 vector, proliferation was significantly suppressed in the corresponding cells transfected with the MT DHCR7 vector (Fig. 7A, B). AGS, MKN28, and HGC-27 cells transfected with the MT DHCR7 vector showed decreased migration and invasion abilities (Fig. 7C). In addition, we performed an apoptosis assay to evaluate the influence of MT DHCR7 on GC cell apoptosis. The results showed that MT DHCR7 significantly increased the apoptosis rate of AGS, MKN28, and HGC-27 cells (Fig. 7D). Furthermore, the cholesterol level in AGS, MKN28, and HGC-27 cells was significantly decreased after transfection with MT DHCR7 (Fig. 7E, F). Taken together, our findings indicated that in GC cells, DHCR7 with rs104886038 and rs104886035 co-mutation decreased cholesterol synthesis; suppressed proliferation, invasion, and migration; and induced apoptosis.

## Discussion

In this work, we used a novel strategy combining genetic information with clinical features, molecular function studies, and amino acid network analysis. Owing to this strategy, we successfully evaluated SNPs identified by a GWAS. Through in vivo and in vitro experiments, DHCR7 was verified to regulate cholesterol biosynthesis and affect proliferation, invasion, migration, and apoptosis in GC cells. Based on protein structure prediction and amino acid network analysis, we identified the mutation sites that significantly affect the stability and function of DHCR7. The verification experiments showed that rs104886038 and rs104886035 co-mutation reduced the protein stability of DHCR7 and induced its degradation through the ubiquitin–proteasome pathway. Mutant DHCR7 lost its biological function, weakened the malignant phenotype of GC cells and further induced apoptosis, indicating potential targets for therapeutic application.

GWASs enable deep sequencing of the whole genome to obtain SNP signatures at various abundance levels and perform correlation analysis with selected features. Earlier GWASs identified genes with a high mutation frequency [11, 27, 28]. Subsequently, some studies have shown that some low-frequency mutations in ATM [12] and SPOCD1 [29] are also highly correlated with the occurrence and development of GC. Moreover, due to the great differences in the genetic backgrounds of populations of different races and from different regions, the prevalence of high-risk genotypes for the same disease differs significantly, especially for cancers such as GC [9]. This implies that although many SNPs have been identified, few of them could be further applied for clinical diagnosis and treatment, which further calls for comprehensive evaluation and verification of GWAS results.

In this work, we enrolled 191 GC patients and 288 healthy volunteers for GWAS analysis and combined these data with some reported potential pathological behavioural factors in GC for a comprehensive analysis. After we combined the data from the training and validation sets, a total of 3 significantly different loci were identified. All three were reported for the first time in GC. Therefore, we first evaluated the risk related to these different genotypes in GC. The results demonstrated that the high-risk genotypes included rs191281603 (G), rs240541 (A), and rs104886038 (A). The combination of these 3 SNPs may exhibit good diagnostic efficiency in GC. Considering that rs191281603 and rs240541 are located in intronic regions of CFHR5 and DCPS, respectively, rs104886038, which is located in the coding sequence of DHCR7, was further studied. We focused on the association of rs104886038 and its genotypes with clinicopathological parameters. First, we found that the risk of some common behavioural factors was not completely consistent between patients with different genotypes. In addition to age, smoking, and *H. pylori* infection as the common risk factors, alcohol consumption seems to be a specific prominent risk factor in rs104886038 (A/A) patients. In some studies of alcoholic fatty liver disease, alcohol was found to cause intracellular cholesterol accumulation and mitochondrial dysfunction [30, 31]. In the rs104886038 (A/G) population, the promoting effect of alcohol consumption on cholesterol accumulation may also be attenuated due to mutations that impair endogenous cholesterol biosynthesis. However, we did not observe significant differences in plasma free cholesterol levels between patients with different genotypes (Figure S4), possibly due to the following reasons: ① We observed that all samples used in this study were heterozygous for the rs104886038 mutation. Compared with the pathogenic loci that cause abnormal growth and development in humans with Smith-Lemli-Opitz syndrome (SLOS) [32–34], the rs104886038 mutation may have a limited effect on the protein and its function. ② In humans, the plasma cholesterol level is affected by dietary cholesterol intake, liver cholesterol synthesis, and cholesterol utilization in tissues and organs; thus, it should not be measured by the change in a single SNP site. ③ Regarding alcohol consumption by participants in this work, some studies have suggested that alcohol consumption can lead to an increase in the plasma LDL level accompanied by a decrease in the HDL level [35], which may result in various levels of plasma free cholesterol. Under the combined action of many factors, the plasma cholesterol level is not determined only by the level of endogenous synthesized cholesterol in cells. In addition, analysis of multiple datasets from TCGA and GEO confirmed the upregulated status of DHCR7 gene expression in GC patients. The gene cluster coexpressed with DHCR7 contains SQLE (*r* = 0.62, *P* = 7.20E-38), SREBP2 (*r* = 0.53, *P* = 9.89E-26), HMGCR (*r* = 0.52, *P* = 3.32E-25) and other key molecules involved in a series of cholesterol synthesis pathways. It is suggested that in GC, cholesterol biosynthesis pathway signalling may be closely related to the risk of disease.

As cholesterol is an important member of the sterol synthesis pathway and an essential molecule constituting cellular and subcellular membranes, its biosynthesis, metabolism, transport and transformation are involved throughout the cellular life cycle [36–39]. Aberrant cholesterol metabolism is involved in the progression of various cancers, including breast cancer [40], pancreatic cancer [41], and GC [42]. DHCR7, a key enzyme in the cholesterol biosynthesis pathway, directly catalyses the conversion of 7-dehydrocholesterol to synthesize cholesterol through the Kandutsch–Russell and Bloch pathways [26]. Importantly, terbinafine, targeting the cholesterol biosynthesis pathway, is gradually being tried and promoted in cancer therapy [43, 44]. In the current study, DHCR7 was identified through a GWAS and clinical analysis. We found that overexpression of DHCR7 increased the cellular cholesterol level and further enhanced the malignant phenotypes of GC cells. In addition, both knockdown of and key mutations in DHCR7 suppressed malignant phenotypes of GC cells through inhibiting cholesterol biosynthesis, which indicated that intervention with the cholesterol synthesis pathway is also a potential treatment strategy for GC. In addition to the molecular target identification provided by traditional functional studies, we tried to explore much more precise therapeutic targets by combining genetic background and amino acid network analyses. GWAS analysis indicated that the L68P mutation (rs104886038) indicated a decreased risk of GC. In fact, limited by the size of the cohort and limitation of GWAS itself, the genetic information we found in GWAS usually tends to be common and mild variants rather than those rare but lethal [45, 46]. And changes at a single SNP locus do not determine whether patients eventually develop GC or their prognostic outcomes after diagnosis. That is the reason why those SNPs found in previous GWAS were unable to be further applicated in clinical. Through structure prediction and amino acid network analysis, we carried out a deeper exploration of the potentially valuable SNP sites that we screened. After combining the co-mutation we predicted in protein structure analysis, we found that co-mutating with rs104886035 (ΔΔGL68P + P51S: -2.9 kcal/mol)could enhance the effect of a single mutation of rs104886038 (ΔΔGL68P: -0.36 kcal/mol), which lead to a sharp decrease in the stability of the DHCR7 protein and eventually induce its degradation by the proteasome system. Under this condition, the cholesterol level in GC cells also decreased significantly, and a series of malignant phenotypes, such as proliferation, invasion and migration, were inhibited. Most importantly, apoptosis was induced in GC cells by this co-mutation. Although we did not generate a cell line with the L68P (rs104886038) mutation alone, the above work undoubtedly provides an important direction for the design of drugs targeting DHCR7. In addition, this is a hypothesis for and attempt at clinical interventions for GC patients with different clinical backgrounds.

This integrated multi-dimensional strategy we provided can be very effective for the in-depth study and application of coding region mutations obtained from GWAS. This strategy can comprehensively considering genetic information, clinicopathological characteristics, and functions of mutated genes, so as to further explain the function of gene polymorphism in GC and explore potential intervention targets. Yet the limitation of this strategy might be the interpretation of mutations in non-coding region. In fact, the results of GWAS usually tend to find a large number of non-coding sequence mutations. It is currently believed that these mutations also play an important role in cancer. For example, mutations in promoter and enhancer regions will affect gene expression [47, 48]. In addition, non-coding RNAs and their binding site mutations are also important pathways for cancer pathogenesis [49–51]. Noncoding RNAs are currently considered to have potential value in the diagnosis and treatment of cancers [52, 53]. The functional annotation and validation of non-coding sites and the study of related non-coding RNAs may be a further supplement to this strategy.

## Conclusion

Overall, this study combined the genetic information obtained via a GWAS with clinical investigation, molecular function studies and amino acid network analysis and eventually showed that DHCR7 regulates cholesterol biosynthesis in GC and that its key co-mutation sites are potential targets for GC therapy. The integrated multi-dimensional analysis strategy that we proposed may be a potential direction for applying genetic background information to clinical diagnosis and mechanistic research.

## Supplementary Information

**Additional file 1: Table S1.** Clinical-pathological features of 191 GC patients and baseline information of 288 healthy controls.

**Additional file 2: Table S2.** Pre-processing steps to assemble the final dataset for GWAS.

**Additional file 3: Figure S1.** Quantile-quantile plot of observed vs. expected - log10(P) scores in GWAS.

**Additional file 4: Table S3. **Target sequence of DHCR7 siRNA.

**Additional file 5: Figure S2. **Map of shuttle and packaging plasmids for lentivirus construction.

**Additional file 6: Table S4.** Antibody information.

**Additional file 7:** **Table S5.** Parameters of logistic regression model including three SNP sites.

**Additional file 8: Figure S3. **Cytotoxicity assay of TAM and AY 9944.

**Additional file 9:** **Figure S4.** Plasma total cholesterol level of GC patients with different genotypes.

## Data Availability

These data were derived from the following resources available in the public domain: 1. RNA-seq data with the corresponding clinical data for 343 GC tissues and 30 adjacent tissues were downloaded from the TCGA database: https://cancergenome.nih.gov/ 2. The DSE66229 dataset was downloaded from the NCBI GEO database: https://www.ncbi.nlm.nih.gov/geo/ 3. Mutation sites in DHCR7 were obtained from the UniProt database: https://www.uniprot.org/uniprot/Q9UBM7

## Abbreviations

AJCC: American Joint Committee on Cancer
ATCC: American Type Culture Collection
AUC: Area Under Curve
CHEH: Cholesterol epoxide hydrolase
CHX: Cycloheximide
CI: Confidence intervals
CQ: Chloroquine
FBS: Foetal bovine serum
GC: Gastric cancer
GWAS: Genome-wide association study
*H.pylori*: *Helicobacter pylori*
HC: Healthy control
HE: Hematoxylin and eosin
IHC: Immunohistochemistry
KEGG: Kyoto Encyclopedia of Genes and Genomes
MAF: Minor allele frequency
MT: Mutation
OR: Odds radio
PALM: Probabilistic natural mapping
ROC: Receiver operating characteristic curve
RT: Room temperature
shRNA: Short hairpin RNA
SLOS: Smith-Lemli-Opitz syndrome
SNP: Single nucleotide polymorphism
TAM: Tamoxifen
WT: Wild-type
