# Supplementary material for: Integrated multi-dimensional analysis highlights DHCR7 mutations involving in cholesterol biosynthesis and contributing therapy of gastric cancer
Source: J Exp Clin Cancer Res. 2023 Jan 30;42:36. doi: 10.1186/s13046-023-02611-6 (PMC9885627; doi:10.1186/s13046-023-02611-6)
Supplement: Supplementary file 1 — Additional file 1: Table S1. Clinical-pathological features of 191 GC patients and baseline information of 288 healthy controls. [file 13046_2023_2611_MOESM1_ESM.pdf]

Tabel S1 Clinical-pathological features of 191 GC patients and baseline information of 288 healthy controls.

sample ID	rs104886038 genotype	GC（GC, 1; HC, 0）	Gender (Male = A, Female = B	Age≥60A＜60B	Smoke（≥10 cigarettes/day, A; <10 cigarettes/day, B)	Heavy drinking (Heavy drinking, A; Others, B)	White meat preference (Yes, A; No, B)	Pickled food preferences (Yes, A; No, B)	H.pylori infect (Yes, A; No, B)	T stage(1-2, A; 3-4, B)	N stage (1-3, A; 0, B)	N stage (1-3, A; 0, B)	TNM(AJCC8) stage(1-2, A; 3-4, B)	Early GC,A; Advanced GC, B	Differentiation (poorly differentiated and undifferentiated, A;Medium to high differentiation, B)	Lauren type(diffused, A; Intesitinal, B)
OG170251034	A/A	1	A	A	B	A	B	B	B	B	A	B	B	2	A	B
OG180250170	A/G	1	A	B	A	B	B	A	B	A	B	B	A	1	N.A	B
OG180250365	A/A	1	A	A	B	A	A	B	B	B	A	B	B	2	A	B
OG180250497	A/A	1	B	B	B	B	B	B	B	B	A	B	B	2	N.A	A
OG180250502	A/A	1	A	A	B	B	B	A	A	A	B	B	A	1	B	B
OG180250512	A/A	1	A	A	B	A	B	A	B	A	A	B	A	1	N.A	B
OG180250514	A/A	1	B	A	B	B	A	B	A	B	N.A	A	B	2	A	B
OG180250519	A/A	1	A	A	B	B	B	A	A	B	A	B	B	2	A	B
OG180250522	A/A	1	A	A	B	A	B	B	A	A	B	B	A	1	B	B
OG180250527	A/A	1	A	A	B	A	B	B	A	B	A	B	B	2	A	B
OG180250529	A/A	1	B	B	B	B	B	A	B	B	A	B	B	2	A	B
OG180250530	A/A	1	A	A	B	A	B	B	B	B	A	B	B	2	B	B
OG180250531	A/A	1	A	B	A	B	B	A	B	A	B	B	A	1	A	B
OG180250532	A/A	1	A	A	A	A	B	A	B	B	A	B	B	2	N.A	B
OG180250533	A/A	1	A	A	A	A	B	B	B	N.A	N.A	A	B	2	N.A	B
OG180250534	A/A	1	A	A	B	B	B	A	B	B	B	B	A	2	N.A	B
OG180250536	A/A	1	A	B	B	B	A	A	A	B	B	B	A	2	A	B
OG180250538	A/A	1	A	A	A	A	B	B	B	A	B	B	A	1	A	B
OG180250539	A/A	1	A	B	A	B	B	A	B	B	A	B	B	2	B	B

OG180250545	A/A	1	A	A	A	B	B	A	B	A	A	B	A	2	A	B
OG180250546	A/A	1	A	A	B	B	B	A	A	A	B	B	A	1	A	B
OG180250547	A/A	1	A	A	B	B	B	A	B	B	A	B	B	2	A	B
OG180250748	A/A	1	B	A	B	B	B	A	A	B	A	B	B	2	A	B
OG180250751	A/A	1	A	A	B	B	B	A	A	B	A	B	B	2	A	B
OG180250752	A/A	1	A	A	B	A	A	B	B	B	A	B	B	2	A	B
OG180250754	A/A	1	A	B	A	A	B	A	A	B	B	B	A	2	B	B
OG180250755	A/A	1	A	A	A	A	A	A	A	B	A	B	B	2	N.A	B
OG180250756	A/A	1	A	A	B	B	B	B	B	N.A	N.A	A	B	2	N.A	A
OG180251145	A/A	1	A	A	A	B	B	B	B	B	A	B	B	2	A	B
OG180251146	A/G	1	A	A	A	A	B	A	A	B	A	B	B	2	A	A
OG180251147	A/A	1	A	B	B	B	B	A	A	A	B	B	A	1	N.A	B
OG180251148	A/G	1	B	A	B	B	B	A	A	B	A	B	A	2	A	B
OG180251149	A/A	1	B	B	B	B	B	A	A	A	B	B	A	2	B	B
OG180251150	A/G	1	B	A	B	B	B	A	B	A	B	B	A	1	B	B
OG180251151	A/G	1	A	A	A	A	B	A	B	B	A	A	B	2	A	B
OG180251152	A/G	1	A	A	B	B	B	A	A	N.A	N.A	N.A	B	2	N.A	B
OG180251153	A/G	1	A	A	A	B	B	A	A	B	A	B	B	2	A	B
OG180251154	A/A	1	A	A	B	B	B	A	A	B	A	B	B	2	A	B
OG180251155	A/G	1	A	A	A	A	B	B	A	B	A	B	B	2	A	B
OG180251156	A/A	1	B	B	B	B	B	B	A	N.A	N.A	N.A	N.A	N.A	N.A	B
OG180251157	A/A	1	A	B	B	B	B	B	A	B	A	B	A	2	A	B
OG180251158	A/A	1	A	B	B	B	A	A	A	A	B	B	A	1	B	B
OG180251159	A/A	1	A	B	B	B	B	A	B	B	B	B	A	2	B	B
OG180251160	A/A	1	A	B	B	B	B	A	B	B	B	B	A	2	A	B
OG180251162	A/A	1	B	B	B	B	B	A	A	A	B	B	A	1	A	B
OG180251163	A/A	1	B	B	B	B	B	A	A	A	B	B	A	1	N.A	B
OG180251164	A/A	1	A	B	B	B	B	A	B	B	B	B	A	2	B	B
OG180251165	A/A	1	B	A	B	B	B	A	B	A	B	B	A	1	A	B
OG180251166	A/A	1	A	A	B	B	B	B	B	A	A	B	A	2	B	B
OG180251167	A/A	1	A	A	B	B	B	A	A	B	A	B	B	2	A	B
OG180251168	A/A	1	A	A	B	B	B	A	A	B	A	B	B	2	A	B
OG180251169	A/A	1	A	A	B	A	B	A	B	B	A	B	B	2	N.A	B
OG180251170	A/A	1	A	A	B	B	B	B	B	N.A	N.A	A	B	2	N.A	B
OG180251171	A/A	1	A	A	A	B	B	A	A	B	A	B	B	2	A	B

OG180251172	A/G	1	A	A	A	B	B	A	A	B	A	B	B	2	N.A	B
OG180251173	A/G	1	A	A	B	B	B	A	A	B	B	B	A	2	A	B
OG180251174	A/G	1	A	A	B	B	B	A	A	B	A	B	B	2	A	B
OG180251176	A/A	1	A	A	B	B	B	B	B	B	A	B	B	2	A	B
OG180251177	A/A	1	A	A	A	B	B	B	B	B	A	B	B	2	A	B
OG180251178	A/A	1	A	A	A	A	B	A	B	B	A	B	B	2	A	B
OG180251179	A/A	1	B	A	B	B	B	A	B	A	B	B	A	1	A	B
OG180251180	A/A	1	B	A	B	B	B	B	B	B	A	B	B	2	N.A	B
OG180251181	A/A	1	B	B	B	B	B	A	A	B	A	B	B	2	A	B
OG180251184	A/A	1	A	A	B	B	B	B	B	B	A	B	B	2	N.A	B
OG180251185	A/A	1	A	B	A	B	B	B	A	A	A	B	A	2	A	B
OG180251188	A/A	1	B	B	B	B	B	A	A	B	A	B	B	2	A	B
OG180251189	A/A	1	A	A	A	A	A	A	B	A	B	B	A	1	B	B
OG180251190	A/A	1	A	A	A	B	B	B	B	B	B	B	A	2	A	B
OG180251191	A/A	1	A	A	B	A	B	A	B	B	A	B	A	2	N.A	B
OG180251192	A/A	1	B	A	B	B	B	A	B	B	B	B	A	2	A	B
OG180251193	A/A	1	A	A	B	B	A	B	A	B	A	A	B	2	N.A	B
OG180251194	A/A	1	A	A	A	B	B	A	A	B	A	B	B	2	N.A	B
OG180251195	A/G	1	A	B	A	A	B	B	A	B	A	B	B	2	A	B
OG180251308	A/G	1	A	A	A	B	B	A	A	A	B	B	A	1	A	B
OG180251471	A/A	1	A	B	A	A	B	B	A	B	A	A	B	2	N.A	B
OG180251473	A/A	1	A	A	A	A	B	A	B	B	A	B	B	2	A	B
OG180251474	A/A	1	A	A	B	B	B	A	B	A	B	B	A	1	B	B
OG180251475	A/A	1	A	A	B	B	B	A	B	A	B	B	A	1	B	B
OG180251476	A/G	1	A	A	A	B	B	A	A	B	A	B	B	2	N.A	B
OG180251477	A/G	1	A	A	B	A	B	A	B	B	B	B	A	2	A	B
OG180251478	A/G	1	A	A	B	B	B	B	A	A	A	A	B	2	A	B
OG180251479	A/G	1	A	B	A	A	B	A	B	N.A	N.A	A	B	2	N.A	B
OG180251480	A/G	1	A	A	A	B	B	A	A	B	A	B	B	2	B	B
OG180251481	A/G	1	A	B	A	B	B	A	B	B	B	B	A	2	A	B
OG180251482	A/G	1	B	A	B	B	B	B	B	B	A	A	B	2	N.A	B
OG180251483	A/G	1	A	A	A	B	B	A	B	A	B	B	A	1	B	B
OG180251484	A/A	1	B	A	B	B	B	A	B	B	A	B	B	2	A	B
OG180251485	A/A	1	B	A	B	B	B	A	B	A	B	B	A	1	A	B
OG180251487	A/A	1	A	A	A	A	B	A	B	A	B	B	A	1	B	B

OG180251489	A/A	1	A	A	A	B	B	A	B	A	B	B	A	1	B	B
OG180251490	A/A	1	B	A	B	B	B	A	A	B	A	B	B	2	N.A	B
OG180251491	A/A	1	B	B	B	B	B	A	A	B	A	B	B	2	N.A	B
OG180251492	A/A	1	A	B	A	A	B	A	A	N.A	N.A	A	B	2	N.A	A
OG180251493	A/A	1	B	A	B	B	B	A	B	B	A	B	B	2	A	B
OG180251494	A/A	1	A	A	A	A	B	B	A	B	A	B	B	2	B	B
OG180251495	A/A	1	B	A	B	B	B	B	B	A	B	B	A	2	B	B
OG180251496	A/A	1	A	A	B	B	B	A	B	B	B	B	A	2	A	B
OG180251497	A/A	1	A	B	B	A	B	B	B	B	B	B	A	2	B	B
OG180251499	A/A	1	A	A	B	B	B	A	B	A	A	B	A	1	B	B
OG180251500	A/A	1	A	B	B	B	B	A	B	A	B	B	A	1	B	B
OG180251503	A/A	1	A	A	B	B	B	B	B	A	B	B	A	2	N.A	B
OG180251504	A/A	1	A	A	B	B	B	A	B	A	A	B	A	1	A	B
OG180251505	A/A	1	A	B	A	A	B	B	B	B	A	B	B	2	N.A	B
OG180251506	A/A	1	B	A	B	B	B	A	B	B	A	B	B	2	A	B
OG180251507	A/A	1	B	B	B	B	B	A	A	B	A	B	B	2	A	B
OG180251509	A/A	1	A	A	A	B	B	A	B	B	A	B	B	2	A	B
OG180251510	A/A	1	A	A	B	B	B	A	A	B	A	B	B	2	A	A
OG180251511	A/A	1	A	A	B	B	B	A	B	B	A	B	A	2	A	B
OG180251512	A/A	1	B	A	B	B	B	A	B	B	A	B	A	2	A	B
OG180251513	A/A	1	A	A	B	B	B	A	B	B	N.A	A	B	2	N.A	B
OG180251514	A/A	1	B	B	B	B	B	A	B	B	B	B	A	2	B	B
OG180251515	A/A	1	B	A	B	B	B	A	B	B	N.A	B	B	2	N.A	B
OG180251516	A/A	1	B	A	B	B	B	A	A	B	A	B	B	2	N.A	B
OG180251517	A/A	1	A	A	A	B	B	B	B	A	B	B	A	1	B	B
OG180251518	A/A	1	A	A	B	B	B	A	B	B	A	B	A	2	A	B
OG180251519	A/A	1	A	A	B	A	B	B	B	B	A	B	B	2	A	B
OG180251521	A/A	1	A	B	A	A	B	A	B	B	A	B	B	2	N.A	B
OG180290422	A/A	1	A	A	B	B	B	A	A	B	B	B	A	2	N.A	B
OG180290423	A/A	1	B	A	B	B	B	B	A	A	B	B	A	1	B	B
OG180290424	A/A	1	A	A	A	B	B	A	B	B	A	B	B	2	A	B
OG180290425	A/A	1	B	B	B	B	A	A	A	B	A	B	B	2	A	B
OG180290426	A/A	1	A	A	B	B	B	B	B	A	A	B	A	2	N.A	B
OG180290427	A/A	1	A	A	A	B	B	B	A	B	A	B	B	2	A	B
OG180290428	A/A	1	A	A	B	A	B	A	B	A	A	B	A	1	A	B

OG180290429	A/A	1	A	A	B	B	B	B	A	B	A	B	B	2	A	B
OG180290430	A/A	1	A	A	B	B	B	B	A	A	A	B	A	1	A	B
OG180290435	A/A	1	B	B	B	B	B	A	B	A	B	B	A	1	A	B
OG180290436	A/A	1	B	A	B	B	B	A	B	B	B	B	A	2	N.A	B
OG180290437	A/A	1	A	B	B	B	B	A	B	A	B	B	A	2	B	B
OG180290440	A/A	1	A	A	A	B	B	A	A	A	B	B	A	1	B	B
OG180290442	A/A	1	A	A	A	A	A	A	B	B	A	B	B	2	B	B
OG180290443	A/A	1	A	A	A	A	B	B	B	B	N.A	A	B	2	N.A	B
OG180290445	A/A	1	A	A	A	A	B	A	A	B	A	B	B	2	N.A	B
OG180290446	A/A	1	B	B	B	B	A	B	B	A	A	B	A	2	A	B
OG180290447	A/A	1	B	A	B	B	B	A	A	A	B	B	A	2	A	B
OG180290448	A/A	1	B	B	B	B	B	A	B	A	B	B	A	1	N.A	B
OG180290449	A/A	1	A	A	B	B	B	A	A	B	A	B	B	2	A	B
OG180290450	A/A	1	A	A	B	B	B	A	B	A	B	B	A	1	N.A	B
OG180290451	A/A	1	B	A	B	B	B	A	A	N.A	N.A	A	B	2	N.A	B
OG180290452	A/A	1	A	A	A	B	B	A	A	B	N.A	N.A	B	N.A	N.A	B
OG180290453	A/A	1	A	A	B	B	B	B	A	A	B	B	A	N.A	B	B
OG180290454	A/A	1	A	A	A	B	B	A	B	B	A	B	B	2	A	B
OG180290456	A/A	1	A	B	A	B	B	A	B	B	A	B	B	2	A	B
OG180290457	A/A	1	B	A	B	B	B	A	B	B	A	B	B	2	A	B
OG180290458	A/A	1	A	A	B	B	B	A	B	N.A	N.A	A	B	2	A	B
OG180290459	A/A	1	B	A	B	B	B	B	B	B	B	B	A	2	N.A	B
OG180290460	A/A	1	B	B	B	B	B	A	B	A	B	B	A	1	A	B
OG180290461	A/A	1	A	A	B	B	B	A	A	A	B	B	A	2	B	B
OG180290462	A/A	1	A	A	A	B	B	A	A	B	A	B	B	2	N.A	B
OG180290463	A/A	1	B	B	A	B	B	A	B	B	A	B	B	2	A	B
OG180290464	A/A	1	B	A	B	B	B	A	B	B	A	B	B	2	A	B
OG180290465	A/A	1	A	B	A	A	B	A	B	A	B	B	A	2	A	B
OG180290466	A/A	1	B	A	B	B	B	B	B	B	B	B	A	2	A	B
OG180290467	A/A	1	B	A	B	B	B	A	B	B	A	B	B	N.A	N.A	B
OG180290469	A/A	1	A	A	B	B	B	A	B	B	A	B	B	2	A	B
OG180290470	A/A	1	A	A	B	B	B	A	A	B	A	A	B	2	A	B
OG180290471	A/A	1	B	A	B	B	B	A	B	B	A	B	B	2	A	B
OG195710791	A/A	1	A	A	A	B	B	B	B	B	A	B	B	2	A	A
OG195710793	A/A	1	A	A	B	B	B	B	B	A	B	B	A	1	A	A

OG195710794	A/A	1	A	A	B	B	B	A	B	B	A	B	A	2	A	B
OG195710795	A/A	1	A	B	B	B	B	A	A	B	A	B	B	2	B	B
OG195710796	A/A	1	A	B	B	A	B	A	B	B	N.A	A	B	2	N.A	B
OG195710797	A/A	1	B	A	B	B	B	A	B	B	A	B	A	2	A	B
OG195710798	A/A	1	B	A	B	B	B	B	B	B	A	B	B	2	N.A	B
OG195710799	A/A	1	A	A	A	A	B	B	B	A	B	B	A	1	A	B
OG195710801	A/A	1	A	A	B	B	B	A	A	B	A	B	A	2	A	B
OG195710802	A/A	1	A	A	A	A	B	A	A	B	X	A	B	2	N.A	B
OG195710803	A/A	1	A	A	A	A	B	B	A	B	B	B	B	2	N.A	A
OG195710804	A/A	1	A	A	B	A	B	A	A	B	B	B	A	2	A	B
S000001	A/A	0	B	B	B	B	A	A	B	A	B	B	A	1	N.A	B
S000005	A/G	0	A	A	B	B	A	A	B	B	B	B	A	2	A	B
S000006	A/A	0	B	B	B	B	B	A	B	B	A	B	B	2	N.A	B
S000007	A/A	0	A	A	A	B	A	A	B	B	A	B	B	2	A	B
S000009	A/G	0	B	B	B	B	B	A	B	B	N.A	B	B	2	N.A	B
S000010	A/A	0	A	B	B	B	A	A	B	B	B	B	A	2	A	B
S000011	A/A	0	A	B	B	B	A	A	A	B	B	B	A	2	B	B
S000012	A/G	0	A	B	A	A	A	B	B	A	B	B	A	1	B	B
S000013	A/A	0	B	B	B	B	B	A	A	A	B	B	A	1	B	B
S000014	A/A	0	A	B	B	B	A	B	B	B	N.A	A	B	2	N.A	B
S000015	A/A	0	A	B	B	B	B	A	A	N.A	N.A	A	B	2	A	B
S000016	A/G	0	A	B	B	B	B	B	B	B	A	B	B	2	N.A	B
S000017	A/A	0	A	B	B	B	A	A	B	B	A	B	B	2	A	B
S000018	A/A	0	B	B	B	B	A	B	B	B	A	B	B	2	A	B
S000019	A/A	0	B	B	B	B	A	A	B	A	B	B	A	1	B	B
S000020	A/A	0	B	A	B	B	B	A	B	A	B	B	A	2	N.A	B
S000021	A/A	0	B	A	B	B	A	A	A	B	N.A	A	B	2	N.A	A
S000022	A/G	0	B	B	B	B	A	A	B	B	A	B	B	2	N.A	B
S000023	A/A	0	B	B	B	B	A	A	A	A	B	B	A	1	B	B
S000024	A/A	0	B	A	B	B	B	B	B	B	A	B	B	2	A	B
S000025	A/G	0	B	B	B	B	A	A	B	B	A	A	B	2	A	B
S000026	A/G	0	B	A	B	B	B	B	B	A	B	B	A	1	B	B
S000027	A/A	0	A	B	A	A	B	B	B	N.A	N.A	N.A	N.A	N.A	N.A	N.A
S000028	A/G	0	B	B	B	B	A	A	B	N.A	N.A	N.A	N.A	N.A	N.A	N.A
S000029	A/A	0	B	B	B	B	B	A	B	N.A	N.A	N.A	N.A	N.A	N.A	N.A

S000030	A/A	0	A	B	B	B	B	A	B	N.A	N.A	N.A	N.A	N.A	N.A	N.A
S000031	A/A	0	A	B	B	A	B	B	B	N.A	N.A	N.A	N.A	N.A	N.A	N.A
S000032	A/A	0	A	B	B	B	B	A	B	N.A	N.A	N.A	N.A	N.A	N.A	N.A
S000033	A/A	0	A	B	A	A	A	A	A	N.A	N.A	N.A	N.A	N.A	N.A	N.A
S000034	A/G	0	B	B	B	B	A	A	A	N.A	N.A	N.A	N.A	N.A	N.A	N.A
S000035	A/A	0	A	B	B	B	A	A	B	N.A	N.A	N.A	N.A	N.A	N.A	N.A
S000036	A/G	0	B	B	B	B	B	B	B	N.A	N.A	N.A	N.A	N.A	N.A	N.A
S000037	A/G	0	B	B	B	B	B	A	B	N.A	N.A	N.A	N.A	N.A	N.A	N.A
S000038	A/A	0	A	B	B	B	A	A	B	N.A	N.A	N.A	N.A	N.A	N.A	N.A
S000039	A/A	0	B	B	B	B	A	A	B	N.A	N.A	N.A	N.A	N.A	N.A	N.A
S000040	A/A	0	A	B	B	B	A	A	B	N.A	N.A	N.A	N.A	N.A	N.A	N.A
S000041	A/A	0	B	B	B	B	A	A	B	N.A	N.A	N.A	N.A	N.A	N.A	N.A
S000042	A/A	0	B	B	B	B	A	A	B	N.A	N.A	N.A	N.A	N.A	N.A	N.A
S000043	A/G	0	A	B	B	A	A	A	B	N.A	N.A	N.A	N.A	N.A	N.A	N.A
S000045	A/A	0	B	B	B	B	A	A	B	N.A	N.A	N.A	N.A	N.A	N.A	N.A
S000046	A/A	0	B	B	B	B	B	A	B	N.A	N.A	N.A	N.A	N.A	N.A	N.A
S000047	A/A	0	B	B	B	B	A	A	B	N.A	N.A	N.A	N.A	N.A	N.A	N.A
S000048	A/G	0	B	B	B	B	A	A	A	N.A	N.A	N.A	N.A	N.A	N.A	N.A
S000049	A/G	0	A	B	B	B	A	B	A	N.A	N.A	N.A	N.A	N.A	N.A	N.A
S000050	A/A	0	A	B	A	B	B	A	B	N.A	N.A	N.A	N.A	N.A	N.A	N.A
S000051	A/A	0	A	A	B	B	B	A	A	N.A	N.A	N.A	N.A	N.A	N.A	N.A
S000052	A/G	0	B	A	B	B	B	A	B	N.A	N.A	N.A	N.A	N.A	N.A	N.A
S000053	A/A	0	A	A	B	B	A	A	A	N.A	N.A	N.A	N.A	N.A	N.A	N.A
S000054	A/A	0	A	B	B	B	B	A	B	N.A	N.A	N.A	N.A	N.A	N.A	N.A
S000055	A/A	0	A	A	B	B	A	A	B	N.A	N.A	N.A	N.A	N.A	N.A	N.A
S000056	A/A	0	A	B	B	B	B	A	B	N.A	N.A	N.A	N.A	N.A	N.A	N.A
S000057	A/A	0	A	A	B	B	B	A	B	N.A	N.A	N.A	N.A	N.A	N.A	N.A
S000058	A/G	0	A	B	A	B	B	B	B	N.A	N.A	N.A	N.A	N.A	N.A	N.A
S000060	A/G	0	A	B	B	B	A	A	B	N.A	N.A	N.A	N.A	N.A	N.A	N.A
S000061	A/A	0	A	B	B	B	A	A	B	N.A	N.A	N.A	N.A	N.A	N.A	N.A
S000064	A/A	0	B	B	B	B	B	A	B	N.A	N.A	N.A	N.A	N.A	N.A	N.A
S000065	A/G	0	A	B	B	A	B	A	B	N.A	N.A	N.A	N.A	N.A	N.A	N.A
S000066	A/G	0	B	B	B	B	B	A	B	N.A	N.A	N.A	N.A	N.A	N.A	N.A
S000067	A/G	0	A	B	B	B	B	A	B	N.A	N.A	N.A	N.A	N.A	N.A	N.A
S000068	A/A	0	A	A	B	B	B	B	A	N.A	N.A	N.A	N.A	N.A	N.A	N.A

S000069	A/A	0	A	B	B	B	B	A	A	N.A	N.A	N.A	N.A	N.A	N.A	N.A
S000070	A/A	0	B	B	B	B	B	A	A	N.A	N.A	N.A	N.A	N.A	N.A	N.A
S000071	A/A	0	B	B	B	B	B	A	B	N.A	N.A	N.A	N.A	N.A	N.A	N.A
S000072	A/A	0	A	A	B	B	B	A	B	N.A	N.A	N.A	N.A	N.A	N.A	N.A
S000073	A/G	0	B	B	B	B	A	A	B	N.A	N.A	N.A	N.A	N.A	N.A	N.A
S000074	A/G	0	B	B	B	B	B	A	B	N.A	N.A	N.A	N.A	N.A	N.A	N.A
S000075	A/G	0	A	B	B	A	B	B	B	N.A	N.A	N.A	N.A	N.A	N.A	N.A
S000076	A/A	0	A	B	B	B	B	A	B	N.A	N.A	N.A	N.A	N.A	N.A	N.A
S000077	A/A	0	B	B	B	B	B	A	B	N.A	N.A	N.A	N.A	N.A	N.A	N.A
S000078	A/A	0	A	B	B	A	B	A	B	N.A	N.A	N.A	N.A	N.A	N.A	N.A
S000079	A/A	0	A	B	B	A	B	A	B	N.A	N.A	N.A	N.A	N.A	N.A	N.A
S000080	A/A	0	A	A	B	B	B	A	A	N.A	N.A	N.A	N.A	N.A	N.A	N.A
S000081	A/G	0	A	A	A	B	B	A	B	N.A	N.A	N.A	N.A	N.A	N.A	N.A
S000082	A/A	0	A	A	B	B	B	A	B	N.A	N.A	N.A	N.A	N.A	N.A	N.A
S000083	A/A	0	B	B	B	B	A	A	B	N.A	N.A	N.A	N.A	N.A	N.A	N.A
S000084	A/A	0	B	B	B	B	A	A	B	N.A	N.A	N.A	N.A	N.A	N.A	N.A
S000085	A/A	0	B	B	B	B	A	A	A	N.A	N.A	N.A	N.A	N.A	N.A	N.A
S000086	A/G	0	B	B	B	B	A	A	B	N.A	N.A	N.A	N.A	N.A	N.A	N.A
S000087	A/G	0	B	B	B	B	A	A	B	N.A	N.A	N.A	N.A	N.A	N.A	N.A
S000088	A/A	0	A	B	B	B	B	A	B	N.A	N.A	N.A	N.A	N.A	N.A	N.A
S000089	A/A	0	A	B	B	A	A	A	B	N.A	N.A	N.A	N.A	N.A	N.A	N.A
S000090	A/A	0	B	B	B	B	A	A	A	N.A	N.A	N.A	N.A	N.A	N.A	N.A
S000091	A/G	0	A	B	B	B	A	A	B	N.A	N.A	N.A	N.A	N.A	N.A	N.A
S000092	A/A	0	A	B	B	B	B	A	B	N.A	N.A	N.A	N.A	N.A	N.A	N.A
S000093	A/G	0	B	B	B	B	B	B	B	N.A	N.A	N.A	N.A	N.A	N.A	N.A
S000094	A/A	0	B	B	B	B	B	A	A	N.A	N.A	N.A	N.A	N.A	N.A	N.A
S000095	A/G	0	B	B	B	B	A	B	B	N.A	N.A	N.A	N.A	N.A	N.A	N.A
S000096	A/A	0	B	B	B	B	B	A	A	N.A	N.A	N.A	N.A	N.A	N.A	N.A
S000097	A/G	0	B	B	B	B	A	A	B	N.A	N.A	N.A	N.A	N.A	N.A	N.A
S000098	A/A	0	A	A	B	B	B	A	B	N.A	N.A	N.A	N.A	N.A	N.A	N.A
S000099	A/G	0	A	B	B	B	B	A	B	N.A	N.A	N.A	N.A	N.A	N.A	N.A
S000100	A/A	0	B	B	B	B	B	A	B	N.A	N.A	N.A	N.A	N.A	N.A	N.A
S000101	A/G	0	A	B	A	A	A	A	A	N.A	N.A	N.A	N.A	N.A	N.A	N.A
S000102	A/A	0	A	B	B	B	B	A	B	N.A	N.A	N.A	N.A	N.A	N.A	N.A
S000103	A/A	0	B	B	B	B	B	B	B	N.A	N.A	N.A	N.A	N.A	N.A	N.A

S000104	A/G	0	A	B	B	A	B	A	B	N.A	N.A	N.A	N.A	N.A	N.A	N.A
S000105	A/A	0	B	B	B	B	A	A	B	N.A	N.A	N.A	N.A	N.A	N.A	N.A
S000106	A/G	0	B	A	B	B	B	A	B	N.A	N.A	N.A	N.A	N.A	N.A	N.A
S000107	A/A	0	B	A	B	B	B	A	A	N.A	N.A	N.A	N.A	N.A	N.A	N.A
S000109	A/G	0	A	B	B	B	B	A	B	N.A	N.A	N.A	N.A	N.A	N.A	N.A
S000110	A/G	0	B	B	B	B	B	A	B	N.A	N.A	N.A	N.A	N.A	N.A	N.A
S000111	A/G	0	B	A	B	B	B	B	B	N.A	N.A	N.A	N.A	N.A	N.A	N.A
S000112	A/G	0	B	B	B	B	B	A	B	N.A	N.A	N.A	N.A	N.A	N.A	N.A
S000113	A/G	0	A	A	B	B	A	A	B	N.A	N.A	N.A	N.A	N.A	N.A	N.A
S000114	A/A	0	B	B	B	B	A	A	B	N.A	N.A	N.A	N.A	N.A	N.A	N.A
S000115	A/A	0	B	A	B	B	A	A	B	N.A	N.A	N.A	N.A	N.A	N.A	N.A
S000116	A/A	0	A	B	A	B	A	A	B	N.A	N.A	N.A	N.A	N.A	N.A	N.A
S000118	A/A	0	B	B	B	B	B	A	B	N.A	N.A	N.A	N.A	N.A	N.A	N.A
S000119	A/G	0	B	B	B	B	A	A	A	N.A	N.A	N.A	N.A	N.A	N.A	N.A
S000120	A/A	0	B	B	B	B	A	A	B	N.A	N.A	N.A	N.A	N.A	N.A	N.A
S000121	A/G	0	B	B	B	B	A	A	B	N.A	N.A	N.A	N.A	N.A	N.A	N.A
S000123	A/A	0	A	A	B	B	A	A	B	N.A	N.A	N.A	N.A	N.A	N.A	N.A
S000124	A/G	0	B	B	B	B	A	A	B	N.A	N.A	N.A	N.A	N.A	N.A	N.A
S000125	A/G	0	A	B	B	A	B	A	B	N.A	N.A	N.A	N.A	N.A	N.A	N.A
S000126	A/A	0	A	B	A	B	B	A	B	N.A	N.A	N.A	N.A	N.A	N.A	N.A
S000127	A/G	0	A	B	B	B	B	A	B	N.A	N.A	N.A	N.A	N.A	N.A	N.A
S000128	A/A	0	A	B	B	B	B	B	B	N.A	N.A	N.A	N.A	N.A	N.A	N.A
S000130	A/A	0	B	B	B	B	B	B	B	N.A	N.A	N.A	N.A	N.A	N.A	N.A
S000131	A/G	0	A	B	B	A	B	B	B	N.A	N.A	N.A	N.A	N.A	N.A	N.A
S000132	A/G	0	B	B	B	B	B	A	B	N.A	N.A	N.A	N.A	N.A	N.A	N.A
S000133	A/G	0	A	B	B	B	B	A	B	N.A	N.A	N.A	N.A	N.A	N.A	N.A
S000134	A/G	0	B	B	B	B	A	A	B	N.A	N.A	N.A	N.A	N.A	N.A	N.A
S000135	A/G	0	B	B	B	B	B	B	B	N.A	N.A	N.A	N.A	N.A	N.A	N.A
S000136	A/A	0	A	A	B	B	A	B	A	N.A	N.A	N.A	N.A	N.A	N.A	N.A
S000137	A/A	0	A	B	B	B	A	A	B	N.A	N.A	N.A	N.A	N.A	N.A	N.A
S000138	A/G	0	B	B	B	B	B	A	B	N.A	N.A	N.A	N.A	N.A	N.A	N.A
S000139	A/A	0	B	B	B	B	A	A	A	N.A	N.A	N.A	N.A	N.A	N.A	N.A
S000140	A/A	0	A	B	B	B	A	A	B	N.A	N.A	N.A	N.A	N.A	N.A	N.A
S000141	A/A	0	B	B	B	B	A	B	A	N.A	N.A	N.A	N.A	N.A	N.A	N.A
S000142	A/G	0	A	A	B	B	A	A	B	N.A	N.A	N.A	N.A	N.A	N.A	N.A

S000143	A/G	0	B	B	A	B	B	A	B	N.A	N.A	N.A	N.A	N.A	N.A	N.A
S000144	A/A	0	A	B	B	B	A	A	B	N.A	N.A	N.A	N.A	N.A	N.A	N.A
S000146	A/A	0	B	A	B	B	B	A	B	N.A	N.A	N.A	N.A	N.A	N.A	N.A
S000147	A/A	0	A	B	B	A	B	A	B	N.A	N.A	N.A	N.A	N.A	N.A	N.A
S000149	A/G	0	B	B	B	B	A	B	B	N.A	N.A	N.A	N.A	N.A	N.A	N.A
S000150	A/G	0	B	B	B	B	B	A	B	N.A	N.A	N.A	N.A	N.A	N.A	N.A
S000151	A/A	0	A	B	A	B	B	A	B	N.A	N.A	N.A	N.A	N.A	N.A	N.A
S000152	A/A	0	A	B	A	A	A	A	B	N.A	N.A	N.A	N.A	N.A	N.A	N.A
S000153	A/G	0	B	B	B	B	B	A	B	N.A	N.A	N.A	N.A	N.A	N.A	N.A
S000154	A/A	0	B	B	B	B	B	B	B	N.A	N.A	N.A	N.A	N.A	N.A	N.A
S000155	A/A	0	A	A	B	B	B	A	B	N.A	N.A	N.A	N.A	N.A	N.A	N.A
S000157	A/A	0	A	B	B	B	B	A	B	N.A	N.A	N.A	N.A	N.A	N.A	N.A
S000158	A/A	0	B	B	B	B	B	A	B	N.A	N.A	N.A	N.A	N.A	N.A	N.A
S000159	A/A	0	B	B	B	B	B	A	B	N.A	N.A	N.A	N.A	N.A	N.A	N.A
S000160	A/A	0	A	B	B	A	B	A	B	N.A	N.A	N.A	N.A	N.A	N.A	N.A
S000161	A/A	0	A	B	A	A	B	B	B	N.A	N.A	N.A	N.A	N.A	N.A	N.A
S000162	A/A	0	B	B	B	B	A	A	B	N.A	N.A	N.A	N.A	N.A	N.A	N.A
S000164	A/A	0	A	B	B	B	B	A	B	N.A	N.A	N.A	N.A	N.A	N.A	N.A
S000165	A/A	0	B	B	A	A	B	A	B	N.A	N.A	N.A	N.A	N.A	N.A	N.A
S000166	A/G	0	B	B	B	B	B	A	B	N.A	N.A	N.A	N.A	N.A	N.A	N.A
S000167	A/G	0	A	B	B	B	B	A	B	N.A	N.A	N.A	N.A	N.A	N.A	N.A
S000168	A/A	0	B	B	B	B	B	A	B	N.A	N.A	N.A	N.A	N.A	N.A	N.A
S000169	A/G	0	B	A	B	B	B	A	B	N.A	N.A	N.A	N.A	N.A	N.A	N.A
S000170	A/A	0	B	A	B	B	B	A	B	N.A	N.A	N.A	N.A	N.A	N.A	N.A
S000171	A/G	0	B	B	B	B	A	A	B	N.A	N.A	N.A	N.A	N.A	N.A	N.A
S000172	A/A	0	A	B	B	B	B	A	B	N.A	N.A	N.A	N.A	N.A	N.A	N.A
S000173	A/G	0	B	B	B	B	B	A	B	N.A	N.A	N.A	N.A	N.A	N.A	N.A
S000174	A/G	0	B	B	B	B	B	A	B	N.A	N.A	N.A	N.A	N.A	N.A	N.A
S000175	A/G	0	A	A	A	A	B	B	B	N.A	N.A	N.A	N.A	N.A	N.A	N.A
S000176	A/A	0	A	B	A	B	B	B	B	N.A	N.A	N.A	N.A	N.A	N.A	N.A
S000177	A/A	0	A	B	A	A	A	A	B	N.A	N.A	N.A	N.A	N.A	N.A	N.A
S000178	A/G	0	A	B	B	B	B	A	B	N.A	N.A	N.A	N.A	N.A	N.A	N.A
S000179	A/A	0	A	B	B	B	A	B	B	N.A	N.A	N.A	N.A	N.A	N.A	N.A
S000180	A/A	0	A	B	B	B	B	B	B	N.A	N.A	N.A	N.A	N.A	N.A	N.A
S000182	A/G	0	B	B	B	B	B	B	B	N.A	N.A	N.A	N.A	N.A	N.A	N.A

S000183	A/A	0	A	B	B	B	B	A	B	N.A	N.A	N.A	N.A	N.A	N.A	N.A
S000185	A/A	0	A	B	A	B	B	A	B	N.A	N.A	N.A	N.A	N.A	N.A	N.A
S000186	A/G	0	B	B	B	B	A	B	B	N.A	N.A	N.A	N.A	N.A	N.A	N.A
S000187	A/G	0	A	A	B	B	B	A	B	N.A	N.A	N.A	N.A	N.A	N.A	N.A
S000188	A/A	0	B	B	B	B	A	A	B	N.A	N.A	N.A	N.A	N.A	N.A	N.A
S000189	A/A	0	A	A	B	B	B	A	A	N.A	N.A	N.A	N.A	N.A	N.A	N.A
S000191	A/A	0	B	B	B	B	B	A	B	N.A	N.A	N.A	N.A	N.A	N.A	N.A
S000192	A/G	0	B	B	B	A	A	A	A	N.A	N.A	N.A	N.A	N.A	N.A	N.A
S000193	A/A	0	B	B	B	B	B	A	A	N.A	N.A	N.A	N.A	N.A	N.A	N.A
S000194	A/G	0	A	B	B	B	B	A	A	N.A	N.A	N.A	N.A	N.A	N.A	N.A
S000195	A/G	0	B	B	B	B	B	B	B	N.A	N.A	N.A	N.A	N.A	N.A	N.A
S000196	A/A	0	A	A	B	B	B	A	B	N.A	N.A	N.A	N.A	N.A	N.A	N.A
S000197	A/A	0	B	B	B	B	A	A	A	N.A	N.A	N.A	N.A	N.A	N.A	N.A
S000198	A/G	0	A	B	B	B	B	A	B	N.A	N.A	N.A	N.A	N.A	N.A	N.A
S000199	A/G	0	A	B	A	A	A	A	A	N.A	N.A	N.A	N.A	N.A	N.A	N.A
S000201	A/G	0	A	A	B	B	B	A	B	N.A	N.A	N.A	N.A	N.A	N.A	N.A
S000202	A/G	0	B	B	B	B	A	A	B	N.A	N.A	N.A	N.A	N.A	N.A	N.A
S000203	A/A	0	B	B	B	B	A	B	B	N.A	N.A	N.A	N.A	N.A	N.A	N.A
S000204	A/A	0	A	A	A	B	B	A	B	N.A	N.A	N.A	N.A	N.A	N.A	N.A
S000205	A/A	0	A	B	B	B	A	A	B	N.A	N.A	N.A	N.A	N.A	N.A	N.A
S000206	A/A	0	A	B	B	B	B	A	A	N.A	N.A	N.A	N.A	N.A	N.A	N.A
S000207	A/A	0	B	A	B	B	B	A	A	N.A	N.A	N.A	N.A	N.A	N.A	N.A
S000208	A/A	0	B	B	B	B	B	B	B	N.A	N.A	N.A	N.A	N.A	N.A	N.A
S000209	A/A	0	B	B	B	B	A	A	B	N.A	N.A	N.A	N.A	N.A	N.A	N.A
S000210	A/A	0	B	A	B	B	A	A	B	N.A	N.A	N.A	N.A	N.A	N.A	N.A
S000211	A/A	0	A	A	B	B	B	A	A	N.A	N.A	N.A	N.A	N.A	N.A	N.A
S000213	A/A	0	A	B	B	A	A	A	B	N.A	N.A	N.A	N.A	N.A	N.A	N.A
S000214	A/A	0	B	B	B	B	A	A	B	N.A	N.A	N.A	N.A	N.A	N.A	N.A
S000215	A/A	0	B	A	B	B	A	A	B	N.A	N.A	N.A	N.A	N.A	N.A	N.A
S000216	A/G	0	B	A	B	B	B	A	B	N.A	N.A	N.A	N.A	N.A	N.A	N.A
S000219	A/G	0	B	B	B	B	A	A	B	N.A	N.A	N.A	N.A	N.A	N.A	N.A
S000220	A/G	0	B	B	B	B	B	A	B	N.A	N.A	N.A	N.A	N.A	N.A	N.A
S000221	A/G	0	B	B	B	B	A	A	B	N.A	N.A	N.A	N.A	N.A	N.A	N.A
S000222	A/G	0	B	B	B	B	B	A	B	N.A	N.A	N.A	N.A	N.A	N.A	N.A
S000223	A/A	0	A	B	B	B	B	A	B	N.A	N.A	N.A	N.A	N.A	N.A	N.A

S000224	A/G	0	B	B	A	B	A	A	B	N.A	N.A	N.A	N.A	N.A	N.A	N.A
S000225	A/A	0	B	B	B	B	B	B	B	N.A	N.A	N.A	N.A	N.A	N.A	N.A
S000226	A/A	0	B	A	B	B	A	A	B	N.A	N.A	N.A	N.A	N.A	N.A	N.A
S000227	A/A	0	B	A	B	B	B	A	B	N.A	N.A	N.A	N.A	N.A	N.A	N.A
S000228	A/A	0	A	B	B	B	B	A	B	N.A	N.A	N.A	N.A	N.A	N.A	N.A
S000229	A/A	0	B	B	B	B	B	A	B	N.A	N.A	N.A	N.A	N.A	N.A	N.A
S000230	A/A	0	B	B	B	B	B	A	B	N.A	N.A	N.A	N.A	N.A	N.A	N.A
S000231	A/G	0	B	B	B	B	B	B	B	N.A	N.A	N.A	N.A	N.A	N.A	N.A
S000232	A/A	0	A	B	A	B	A	A	B	N.A	N.A	N.A	N.A	N.A	N.A	N.A
S000233	A/A	0	A	B	B	B	B	A	B	N.A	N.A	N.A	N.A	N.A	N.A	N.A
S000235	A/A	0	A	B	B	B	B	A	B	N.A	N.A	N.A	N.A	N.A	N.A	N.A
S000236	A/A	0	B	A	B	B	B	A	B	N.A	N.A	N.A	N.A	N.A	N.A	N.A
S000238	A/A	0	B	B	B	B	B	A	B	N.A	N.A	N.A	N.A	N.A	N.A	N.A
S000239	A/A	0	A	B	B	A	B	A	B	N.A	N.A	N.A	N.A	N.A	N.A	N.A
S000240	A/A	0	B	B	B	B	B	A	B	N.A	N.A	N.A	N.A	N.A	N.A	N.A
S000241	A/A	0	A	B	A	A	B	B	B	N.A	N.A	N.A	N.A	N.A	N.A	N.A
S000242	A/A	0	A	B	B	B	B	A	B	N.A	N.A	N.A	N.A	N.A	N.A	N.A
S000243	A/A	0	B	B	B	B	A	A	A	N.A	N.A	N.A	N.A	N.A	N.A	N.A
S000244	A/A	0	A	A	B	B	A	A	B	N.A	N.A	N.A	N.A	N.A	N.A	N.A
S000245	A/A	0	B	B	B	B	A	A	B	N.A	N.A	N.A	N.A	N.A	N.A	N.A
S000247	A/A	0	A	B	A	A	A	A	B	N.A	N.A	N.A	N.A	N.A	N.A	N.A
S000248	A/A	0	B	B	B	B	B	A	A	N.A	N.A	N.A	N.A	N.A	N.A	N.A
S000249	A/A	0	B	B	B	B	B	A	A	N.A	N.A	N.A	N.A	N.A	N.A	N.A
S000251	A/A	0	B	A	B	B	B	B	B	N.A	N.A	N.A	N.A	N.A	N.A	N.A
S000252	A/A	0	A	A	B	A	A	A	B	N.A	N.A	N.A	N.A	N.A	N.A	N.A
S000253	A/A	0	B	A	B	B	A	A	B	N.A	N.A	N.A	N.A	N.A	N.A	N.A
S000254	A/A	0	A	B	B	B	B	A	B	N.A	N.A	N.A	N.A	N.A	N.A	N.A
S000255	A/A	0	A	B	B	B	A	B	A	N.A	N.A	N.A	N.A	N.A	N.A	N.A
S000256	A/G	0	A	B	A	A	A	A	B	N.A	N.A	N.A	N.A	N.A	N.A	N.A
S000258	A/A	0	A	A	B	B	B	A	A	N.A	N.A	N.A	N.A	N.A	N.A	N.A
S000259	A/A	0	B	A	B	B	B	A	A	N.A	N.A	N.A	N.A	N.A	N.A	N.A
S000260	A/A	0	A	B	B	B	B	A	A	N.A	N.A	N.A	N.A	N.A	N.A	N.A
S000261	A/A	0	A	B	B	B	B	A	B	N.A	N.A	N.A	N.A	N.A	N.A	N.A
S000262	A/G	0	B	B	B	B	B	A	B	N.A	N.A	N.A	N.A	N.A	N.A	N.A
S000263	A/A	0	B	B	B	B	A	A	B	N.A	N.A	N.A	N.A	N.A	N.A	N.A

S000264	A/A	0	B	B	B	B	A	A	A	N.A	N.A	N.A	N.A	N.A	N.A	N.A
S000265	A/G	0	B	A	B	B	A	B	B	N.A	N.A	N.A	N.A	N.A	N.A	N.A
S000266	A/G	0	A	B	B	B	B	A	B	N.A	N.A	N.A	N.A	N.A	N.A	N.A
S000267	A/A	0	A	B	A	B	B	A	B	N.A	N.A	N.A	N.A	N.A	N.A	N.A
S000268	A/A	0	B	B	B	B	B	A	B	N.A	N.A	N.A	N.A	N.A	N.A	N.A
S000269	A/G	0	A	B	B	B	A	A	B	N.A	N.A	N.A	N.A	N.A	N.A	N.A
S000270	A/A	0	B	B	B	B	A	A	B	N.A	N.A	N.A	N.A	N.A	N.A	N.A
S000271	A/G	0	A	B	B	B	B	A	A	N.A	N.A	N.A	N.A	N.A	N.A	N.A
S000273	A/A	0	A	B	A	B	A	A	B	N.A	N.A	N.A	N.A	N.A	N.A	N.A
S000274	A/G	0	B	B	B	B	B	A	A	N.A	N.A	N.A	N.A	N.A	N.A	N.A
S000275	A/G	0	B	B	B	B	B	A	B	N.A	N.A	N.A	N.A	N.A	N.A	N.A
S000276	A/A	0	A	B	B	B	A	A	A	N.A	N.A	N.A	N.A	N.A	N.A	N.A
S000277	A/G	0	A	B	A	B	A	A	B	N.A	N.A	N.A	N.A	N.A	N.A	N.A
S000278	A/G	0	B	A	B	B	B	A	B	N.A	N.A	N.A	N.A	N.A	N.A	N.A
S000279	A/G	0	A	B	B	B	A	A	B	N.A	N.A	N.A	N.A	N.A	N.A	N.A
S000280	A/G	0	B	B	B	B	A	A	B	N.A	N.A	N.A	N.A	N.A	N.A	N.A
S000281	A/G	0	B	B	B	B	B	A	B	N.A	N.A	N.A	N.A	N.A	N.A	N.A
S000283	A/A	0	A	B	B	B	B	B	A	N.A	N.A	N.A	N.A	N.A	N.A	N.A
S000284	A/G	0	B	B	B	B	B	B	A	N.A	N.A	N.A	N.A	N.A	N.A	N.A
S000285	A/A	0	A	B	A	B	B	A	B	N.A	N.A	N.A	N.A	N.A	N.A	N.A
S000286	A/A	0	A	B	B	B	A	B	B	N.A	N.A	N.A	N.A	N.A	N.A	N.A
S000287	A/G	0	B	B	B	B	B	B	B	N.A	N.A	N.A	N.A	N.A	N.A	N.A
S000288	A/G	0	A	A	B	B	B	B	B	N.A	N.A	N.A	N.A	N.A	N.A	N.A
