# Supplementary material for: Integrated multi-dimensional analysis highlights DHCR7 mutations involving in cholesterol biosynthesis and contributing therapy of gastric cancer
Source: J Exp Clin Cancer Res. 2023 Jan 30;42:36. doi: 10.1186/s13046-023-02611-6 (PMC9885627; doi:10.1186/s13046-023-02611-6)
Supplement: Supplementary file 2 — Additional file 2: Table S2. Pre-processing steps to assemble the final dataset for GWAS. [file 13046_2023_2611_MOESM2_ESM.pdf]

Table S2 Pre-processing steps to assemble the final dataset for GWAS.

Step	Pre-processing	Sample	SNP	Note
1	initial	200 control	817528	
		150 case		
2	polymorphism check	200 control	779550	exclusion of 37978 non-SNPs
		150 case		
3	sex check	200 control	779550	check the match between self-reported sex and gene-chip-revealed sex
		150 case		
4	SNPs on 22 autosomes	200 control	741007	exclusion of 38543 non-autosome SNPs
		150 case		
5	SNP-level filtering	200 control	261868	exclusion of 479139 SNPs due to low call rate (threshold = 0.95) or low minor allele frequency (threshold = 0.05)
		150 case		
6	sample-level filtering	199 control	261868	exclusion of 1 individuals due to low call rate (threshold = 0.95)
		150 case		
7	ancestry filtering	199 control	261868	no individual excluded given the largely homogeneous Chinese ancestry in PCA
		150 case		
8	HWE filtering	199 control	257746	Exclusion of 4122 SNPs due to the rejection of Hardy-Weinberg Equilibrium at adjusted α of 1×10^{-6}
		150 case		
		**Model**	**Software**	
9	GWAS Training set	Additive model	plink	
	GWAS Training set	Dominant model	plink	
	GWAS Training set	Recessive model	plink	
10	GWAS Validation set	Additive model	plink	
