# Supplementary figures and images for: Integrated multi-dimensional analysis highlights DHCR7 mutations involving in cholesterol biosynthesis and contributing therapy of gastric cancer
Source: J Exp Clin Cancer Res. 2023 Jan 30;42:36. doi: 10.1186/s13046-023-02611-6 (PMC9885627; doi:10.1186/s13046-023-02611-6)

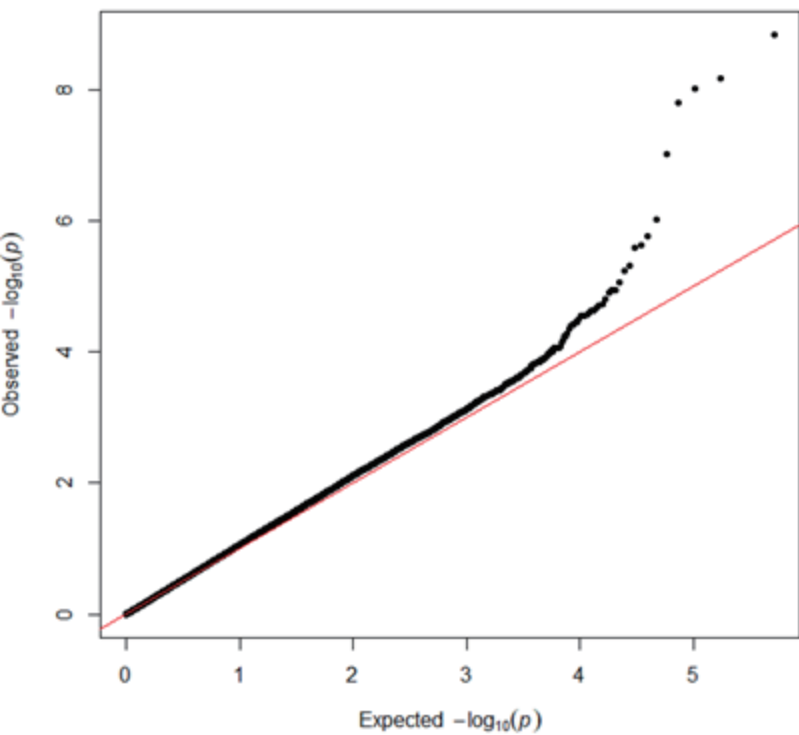

Supplement: Supplementary file 3 — Additional file 3: Figure S1. Quantile-quantile plot of observed vs. expected - log10(P) scores in GWAS. [file 13046_2023_2611_MOESM3_ESM.pdf]

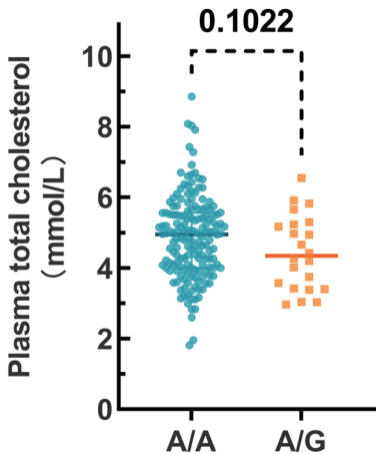

Supplement: Supplementary file 9 — Additional file 9: Figure S4. Plasma total cholesterol level of GC patients with different genotypes. [file 13046_2023_2611_MOESM9_ESM.pdf]
