# Supplementary material for: Integrated multi-dimensional analysis highlights DHCR7 mutations involving in cholesterol biosynthesis and contributing therapy of gastric cancer
Source: J Exp Clin Cancer Res. 2023 Jan 30;42:36. doi: 10.1186/s13046-023-02611-6 (PMC9885627; doi:10.1186/s13046-023-02611-6)
Supplement: Supplementary file 4 — Additional file 4: Table S3. Target sequence of DHCR7 siRNA. [file 13046_2023_2611_MOESM4_ESM.pdf]

Table S3 Target sequence of DHCR7 siRNA

siRNA	Target Sequence
DHCR7-siRNA-1	GTCTAGATGGCGTCACCAA
DHCR7-siRNA-2	AGACTCCACCTATAACGAG
