# Supplementary material for: Integrated multi-dimensional analysis highlights DHCR7 mutations involving in cholesterol biosynthesis and contributing therapy of gastric cancer
Source: J Exp Clin Cancer Res. 2023 Jan 30;42:36. doi: 10.1186/s13046-023-02611-6 (PMC9885627; doi:10.1186/s13046-023-02611-6)
Supplement: Supplementary file 5 — Additional file 5: Figure S2. Map of shuttle and packaging plasmids for lentivirus construction. [file 13046_2023_2611_MOESM5_ESM.pdf]

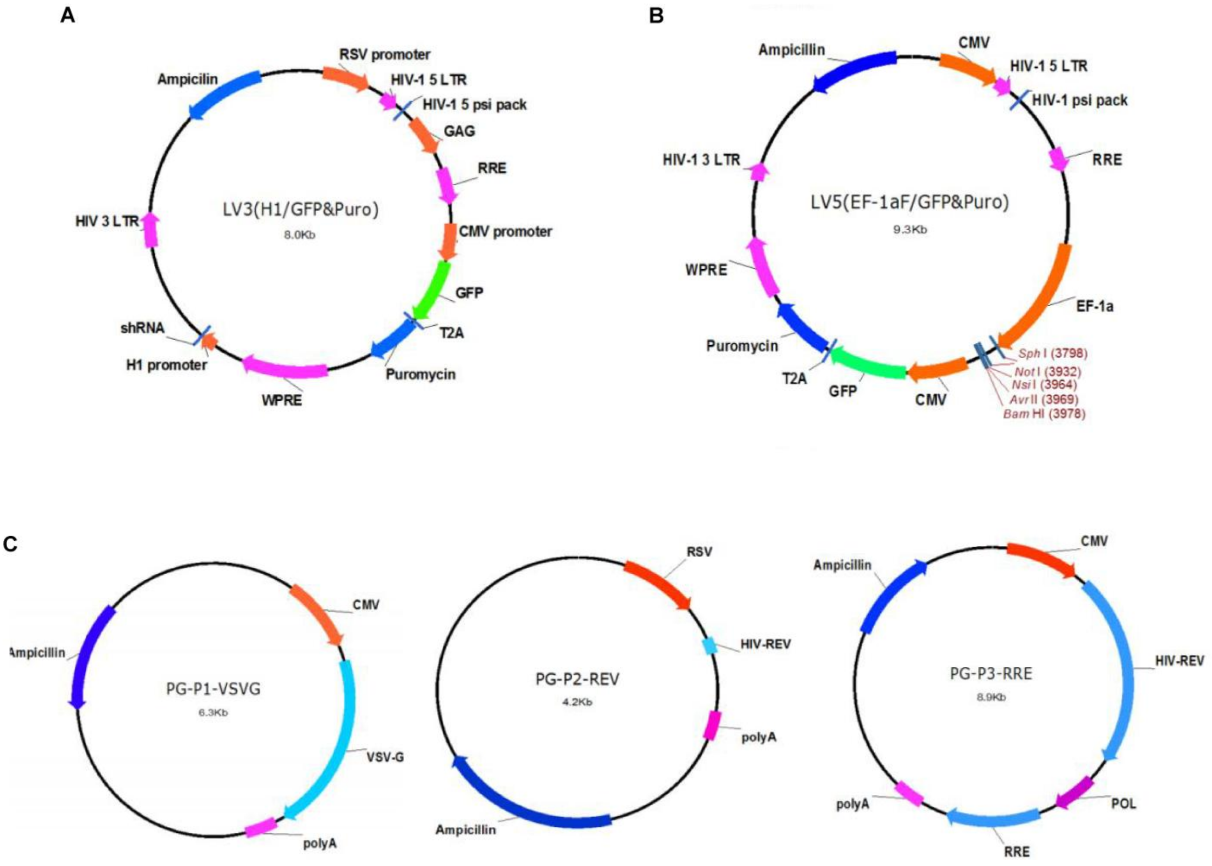

Figure S2 Map of shuttle and packaging plasmids for lentivirus construction. (A) Map of shuttle plasmid carrying shRNA of DHCR7. (B) Map of shuttle plasmid carrying cDNA of DHCR7. (C) Map of packaging plasmid used for lentivirus construction.
