# Supplementary material for: Integrated multi-dimensional analysis highlights DHCR7 mutations involving in cholesterol biosynthesis and contributing therapy of gastric cancer
Source: J Exp Clin Cancer Res. 2023 Jan 30;42:36. doi: 10.1186/s13046-023-02611-6 (PMC9885627; doi:10.1186/s13046-023-02611-6)
Supplement: Supplementary file 6 — Additional file 6: Table S4. Antibody information. [file 13046_2023_2611_MOESM6_ESM.pdf]

Table S4 Antibody information

Antibody	Brand	Cat. No	Dilution Rate	MW (kDa)	Species
DHCR7	Abcam	ab230462	1:1000	54	Rabbit IgG
GAPDH	Proteintech	60004-1-Ig	1:20000	36	Mouse IgG
Ki-67	Proteintech	27309-1-AP	1:2000	359	Rabit IgG
