# Supplementary material for: Integrated multi-dimensional analysis highlights DHCR7 mutations involving in cholesterol biosynthesis and contributing therapy of gastric cancer
Source: J Exp Clin Cancer Res. 2023 Jan 30;42:36. doi: 10.1186/s13046-023-02611-6 (PMC9885627; doi:10.1186/s13046-023-02611-6)
Supplement: Supplementary file 7 — Additional file 7: Table S5. Parameters of logistic regression model including three SNP sites. [file 13046_2023_2611_MOESM7_ESM.pdf]

Table S5 Parameters of logistic regression model including three SNP sites.

	β	S.E.	Wald	Sig.	Exp(β) 95% CI
rs240541 (A/A:0; A/G:1; G/G:2)	-1.27	0.20	42.46	7.22E-11	0.28(0.19-0.41)
rs191281603 (C/C:0; C/G:1; G/G:2)	2.03	0.30	47.01	7.05E-12	7.64(4.27-13.66)
rs104886038 (A/A:0; A/G:1)	-1.70	0.33	25.83	3.73E-07	0.18(0.10-0.35)
factor	0.88	0.27	10.62	1.12E-03	2.41
