# Supplementary material for: Integrated multi-dimensional analysis highlights DHCR7 mutations involving in cholesterol biosynthesis and contributing therapy of gastric cancer
Source: J Exp Clin Cancer Res. 2023 Jan 30;42:36. doi: 10.1186/s13046-023-02611-6 (PMC9885627; doi:10.1186/s13046-023-02611-6)
Supplement: Supplementary file 8 — Additional file 8: Figure S3. Cytotoxicity assay of TAM and AY 9944. [file 13046_2023_2611_MOESM8_ESM.pdf]

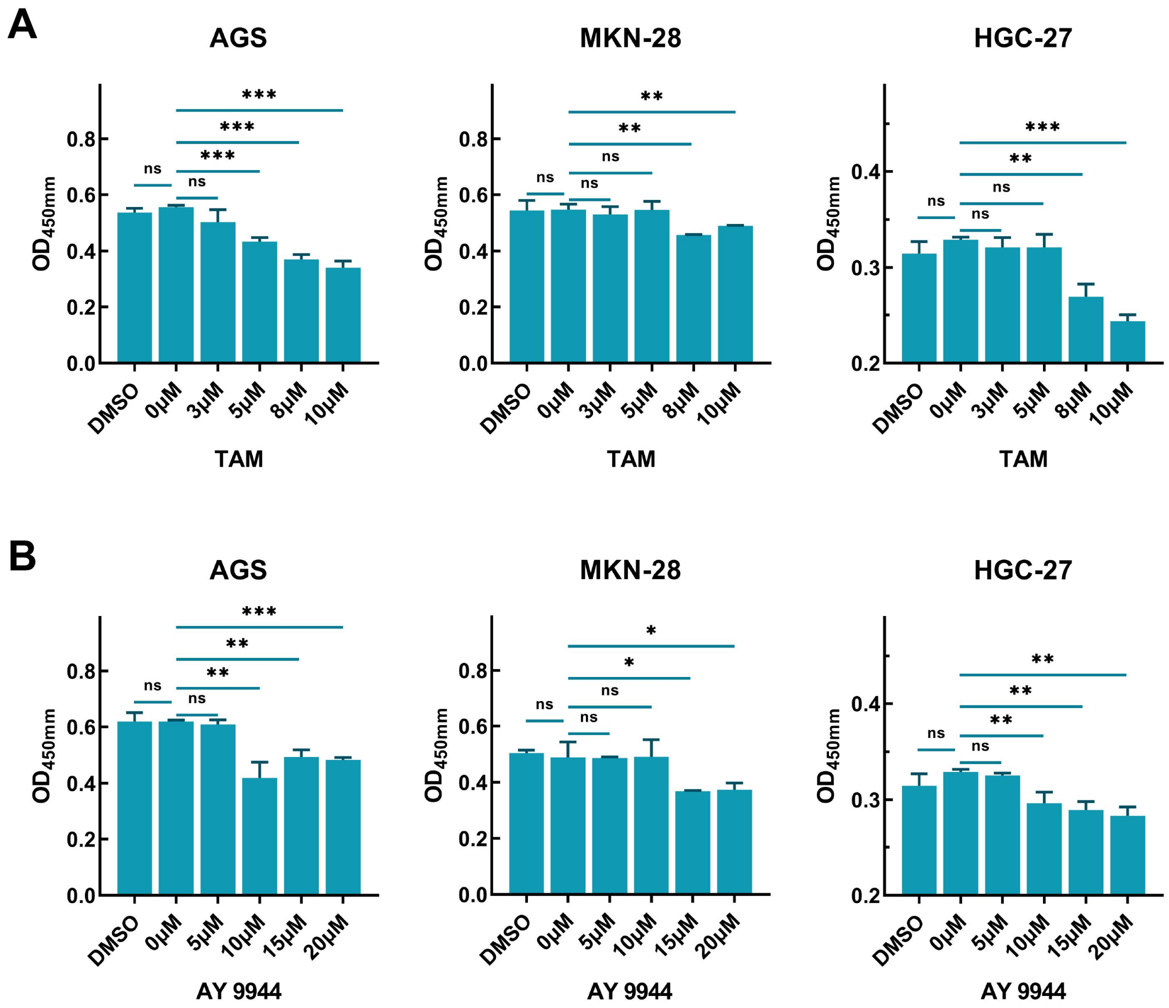

Figure S3 Cytotoxicity assay of TAM and AY 9944. (A) Cytotoxicity assay of TAM. The GC cells were treated with TAM of different concentration (0, 3, 5, 8, 10μM) or DMSO for 24 hours, and the cytotoxicity were measured by CCK8 assay. The highest concentration that did not cause significant cytotoxicity was chosen as the working concentration of TAM. (B) Cytotoxicity assay of AY 9944. The GC cells were treated with AY 9944 of different concentration (0, 5, 10, 15, 20μM) or DMSO for 24 hours, and the cytotoxicity were measured by CCK8 assay. The highest concentration that did not cause significant cytotoxicity was chosen as the working concentration of AY 9944.
